# Discovery of a Novel Polymer for Xeno‐Free, Long‐Term Culture of Human Pluripotent Stem Cell Expansion

**DOI:** 10.1002/adhm.202001448

**Published:** 2020-12-28

**Authors:** Aishah Nasir, Jordan Thorpe, Laurence Burroughs, Joris Meurs, Sara Pijuan‐Galito, Derek J. Irvine, Morgan R. Alexander, Chris Denning

**Affiliations:** ^1^ Division of Cancer & Stem Cells Biodiscovery Institute University of Nottingham Nottingham NG7 2RD UK; ^2^ School of Pharmacy University of Nottingham Nottingham NG7 2RD UK; ^3^ Department of Chemical and Environmental Engineering University of Nottingham Nottingham NG7 2RD UK

**Keywords:** biomaterials discovery, Essential 8 medium, human pluripotent stem cells, polymer microarrays, xeno‐free conditions

## Abstract

Human pluripotent stem cells (hPSCs) can be expanded and differentiated in vitro into almost any adult tissue cell type, and thus have great potential as a source for cell therapies with biomedical application. In this study, a fully‐defined polymer synthetic substrate is identified for hPSC culture in completely defined, xenogenic (xeno)‐free conditions. This system can overcome the cost, scalability, and reproducibility limitations of current hPSC culture strategies, and facilitate large‐scale production. A high‐throughput, multi‐generational polymer microarray platform approach is used to test over 600 unique polymers and rapidly assess hPSC‐polymer interactions in combination with the fully defined xeno‐free medium, Essential 8 (E8). This study identifies a novel nanoscale phase separated blend of poly(tricyclodecane‐dimethanol diacrylate) and poly(butyl acrylate) (2:1 v/v), which supports long‐term expansion of hPSCs and can be readily coated onto standard cultureware. Analysis of cell‐polymer interface interactions through mass spectrometry and integrin blocking studies provides novel mechanistic insight into the role of the E8 proteins in promoting integrin‐mediated hPSC attachment and maintaining hPSC signaling, including ability to undergo multi‐lineage differentiation. This study therefore identifies a novel substrate for long‐term serial passaging of hPSCs in serum‐free, commercial chemically‐defined E8, which provides a promising and economic hPSC expansion platform for clinical‐scale application.

## Graphical abstract

Discovery of poly(tricylodecane‐dimethanol diacrylate‐blend‐butyl acrylate) substrate offers a cost‐effective and xenogenic‐free adherent culture system for human pluripotent stem cells in Essential 8 medium. This scalable substrate can be readily applied to tissue culture plasticware; whilst retaining stem cell integrity after long‐term serial passage. Fulfillment of regulatory culture requirements, makes this a desirable expansion platform for clinical‐scale, therapeutic, and biomedical applications.

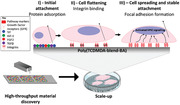

To improve application of adherent human pluripotent stem cell (hPSC) culture, there has been a shift from the use of animal‐derived feeder layers and poorly defined media to systems using surfaces and culture media that are fully‐defined.^[^ ^1^, ^2^, ^3^, ^4^, ^5^, ^6^, ^7^, ^8^, ^9^, ^10^, ^11^, ^12^, ^13^, ^14^, ^15^, ^16^, ^17^, ^18^, ^19^ ^]^ Several commonly utilized feeder‐free hPSC culture systems used with defined media formulations still rely on a broad range of proteins, lipids, small molecules, matrix proteins (e.g. Matrigel, vitronectin, laminin, and fibronectin), or peptide containing surfaces (e.g. Synthemax and PuraMatrix) to promote hPSC culture. Minimizing or eliminating the use of such biological reagents would help to overcome batch‐batch variability during cell processing and reduce costs, which limit large‐scale production (estimated at $10 000‐$15 000 for 1 billion hPSCs required for a single patient intervention).^[^ ^20^ ^]^

Use of polymers as culture substrates whilst safe and cost‐effective solutions identified by us and others have been limited by their dependence on xenogenic (xeno) components from culture medium (e.g. bovine serum albumin containing commercial defined mTESR1 and StemPro) for maintaining hPSC culture.^[^ ^21^, ^22^, ^23^, ^24^, ^25^ ^]^ In the following study, we used high‐throughput microarray screening strategies (previously used by us and others) to identify novel materials capable of supporting hPSCs in a culture system simplified by using the xeno‐free, commercial, chemically‐defined Essential 8 (E8) medium.^[^ ^26^, ^27^, ^28^, ^29^ ^]^ Subsequent characterization of cell interactions with the scaled up synthetic polymer revealed the underlying mechanisms governing cellular response without the contribution of xenogenic components.

Using a multigenerational polymer microarray screening approach, the first generation array consisted of 284 chemically diverse monomers (photo‐curable and readily commercially available) pin‐printed and UV polymerized onto poly(2‐hydroxyethyl methacrylate) (polyHEMA) coated slides as spots in triplicate (**Figure** **1**a, monomer structures presented in Figure S1, Supporting Information with monomer names in Table S1, Supporting Information).^[^ ^21^, ^30^ ^]^ ReBl‐PAT cells, a human induced pluripotent stem cell (hiPSC) line generated in‐house,^[^ ^31^ ^]^ were seeded onto arrays in E8 for 24 h before quantification of cell attachment and response using automated fluorescence microscopy. Materials were ranked by total number of nuclei for cell attachment gauged by DAPI staining (Figure S2a,b Supporting Information, see rank order with polymer names Table S2, Supporting Information), or by number of OCT4+ cells as a marker of pluripotency (Figure S2c, Supporting Information, see rank order with polymer names Table S2, Supporting Information), and the two parameters were plotted against each other (Figure 1b,c).

**Figure 1:**
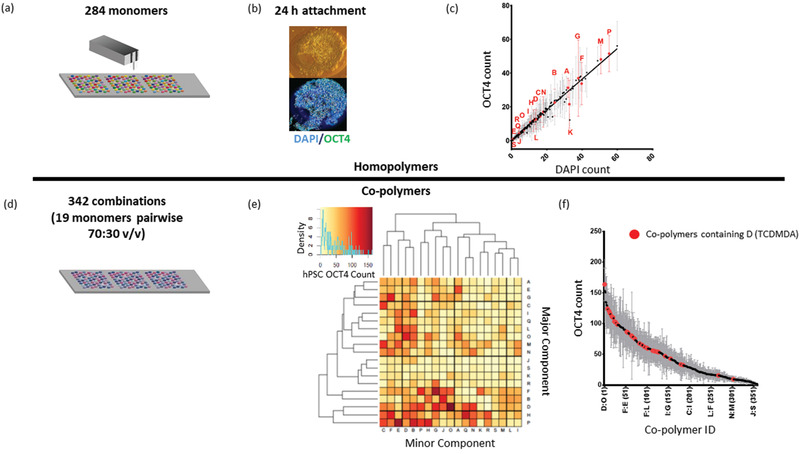
Multi‐generation microarray screen of polymeric substrates a) A first‐generation array of 284 chemically diverse monomers were screened for hPSC attachment with ReBl‐PAT hiPSCs in E8 medium for 24 h. b) Arrays were then fixed and stained for pluripotent marker OCT4, imaged using Imstar automated fluorescence microscopy and OCT4+ nuclei and total nuclei counts assessed with Cell‐Profiler. Representative image shows a polymer spot (*n*) supporting high hPSC attachment. c) Attachment on materials is ranked by OCT4+ nuclei count plotted against total cell number (DAPI). Nineteen materials selected for second‐generation co‐polymer screening (highlighted in red). Each point represents mean (*n* = 9) and SEM for OCT4 count. d) A total of 361 chemistries screened for 24 h included 19 selected monomers printed alone and mixed pairwise (2:1 v/v). e) OCT4+ hPSC attachment (*n* = 9) was clustered by Euclidean distance measure (intensity scale represents OCT4+ nuclei count) and f) ranked (high to low) (full list in Table S3, Supporting Information). Polymer D, (TCDMDA) containing polymers are denoted in red. All letter IDs mentioned are defined in Figure S3, Supporting Information.

From this initial screen, nineteen monomers were selected for a second‐generation co‐polymer screen (denoted in Figure 1c as red letter IDs, and corresponding polymer structure shown in Figure S3, Supporting Information). Monomers residing on the *y* = *x* line show OCT4 expression in >80% hPSCs, whereas those shifted below the line show decreasing percentage of pluripotency either from loss of expression or attachment of non‐viable debris cells. All selected polymers cover a large chemical diversity, show high percentages of OCT4 expression (>80%), but a varied level of attachment. Examples of materials taken forward to the second screen include high attachment, monomer P (tetrahydrofurfuryl acrylate, THFuA; ≈55 ± 35 cells spot^−1^, 93% OCT4 expression) to low attachment monomer S (*N*‐(hydroxymethyl)acrylamide, HMAm; ≈1 ± 2 cells spot^−1^, 100% OCT4 expression). This panel of candidates provided a variety of polymer combinations to be assessed in a second generation screen, where homopolymers were included alone and mixed pairwise as 2:1 v/v (where each monomer was combined as a major and minor component) prior to printing to create a library of 361 chemistries printed in triplicate (Figure 1d).

Screening 361 co‐polymers by OCT4+ ReBl‐PAT attachment at 24 h gave a *z* factor ≈ 0.5,^[^ ^32^ ^]^ indicating good assay suitability was calculated from ReBl‐PAT attachment to Matrigel (positive sample; mean = 125 and SD = 18) and polyHEMA (negative control, used for array slide background coating; mean = 5 and SD = 4). Clustering using Euclidean measure distance (Figure 1e) and ranking by (Figure 1f, see Table S3, Supporting Information for polymer names) identified almost 80 chemistries which supported high hPSC attachment (mean cell number > 75 cells spot^−1^, > 90% OCT4+ cells). More than 25% of these polymers contained monomer D, tricylodecane‐dimethanol diacrylate (TCDMDA) as a homopolymer and co‐polymer (combined pairwise as either a major or minor component) (Figure 1f). TCDMDA also supported attachment to human dental pulp‐derived stem cells in previous microarray screening studies as both a homopolymer and co‐polymer.^[^ ^28^ ^]^

To refine the candidate list further, the synergy ratio (SR) of co‐polymer combinations was assessed; a concept we have previously defined where cell response to the co‐polymer is compared to individual responses of their monomer counterparts (microarray screening and data acquisition methods, Supporting Information).^[^ ^27^, ^33^ ^]^ While most (≈70%) of the 342 combinations were antagonistic or merely additive (SR ≤ 1), ≈30% of the co‐polymer features were determined to be synergistic (SR > 1) (Figure S4a,b, Supporting Information). Consistent with previous studies, synergistic combinations were often related to co‐polymers that combined a high attachment monomer with a low attachment monomer. Candidates taken forward for scale‐up were chosen on the basis of high cell attachment scores (at 24 and 48 h time points; **Figure** **2**; Figure S5, Supporting Information), and consideration of synergistic performance.

**Figure 2:**
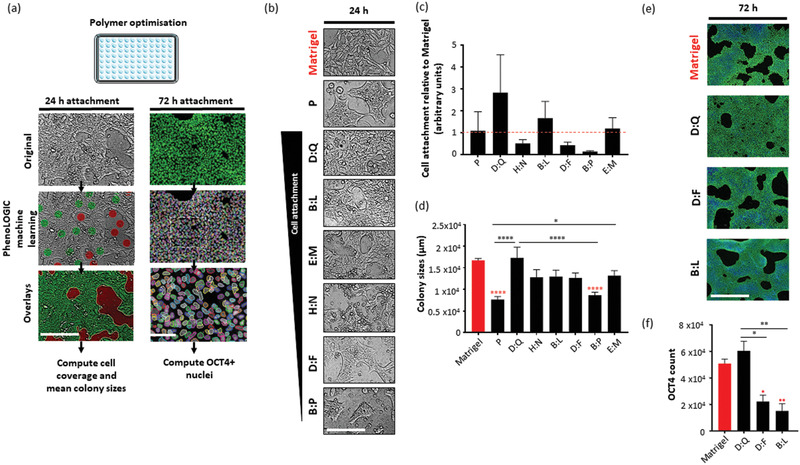
Screening of co‐polymer candidates at scale‐up. a) ReBl‐PAT hPSCs were cultured in E8 on polymers coated on 96 well plates (*n* = 9/condition). Attachment was assessed at 24 and 72 h time‐points and Matrigel was included as a positive control (highlighted in red). All images (5 fields of view per well) were captured (Operetta, Perkin Elmer) and processed using Harmony image analysis software (Perkin Elmer). Cell attachment (cell coverage and mean colony sizes) at 24 h was quantified from brightfield live‐cell images processed using scripts developed with PhenoLOGIC machine learning (script training (left center panel) training: green dots = cells, red dots background, resultant overlays (left bottom panel)) to create a mask for cell coverage per well. At 72 h, hPSCs were fixed and stained for OCT4 expression. OCT4+ nuclei were quantified from fluorescence images (script right panel). b) Representative brightfield images of ReBl‐PATs cultured on polymers (structures to letter IDs in Table S1, Supporting Information) and Matrigel in E8 medium after 24 h. hPSC attachment on co‐polymers are ranked by mean cell coverage/well quantified in c) relative to Matrigel control. (*n* = 9) Co‐polymer array screen ranking denoted in brackets above each bar was taken from total observed (total nuclei count) attachment (Table S3, Supporting Information). d) Mean colony sizes (tightly packed cells with defined outer border) were calculated per field of view (*n* = 15 per condition). Bar graphs represent averaged colony sizes (*n*) of matrigel (164), P (57), D:Q (54), B:L (53), E:M (54), H:N (47), D:F (53), and B:P (62). One way ANOVA followed by Tukey's multiple comparison tests (*p < 0.05,^****^ *p* < 0.0001) were performed. Statistical differences denoted with red asterisks represent comparisons between polymers and Matrigel. Remaining comparisons are labeled. e) Representative images and f) OCT4+ cell attachment per well (*n*) on polymers at 72 h (≈90% OCT4 expression per well). One‐way ANOVA followed by Tukey's multiple comparison tests (*p < 0.05) (*n* = 9). All graphs represent mean (±SEM). Scale bars = 200 µm.

The microarray assays evaluated attachment and retention of pluripotency for 24 h, so our next experiments focused on periods up to 72 h as an indicator of compatibility for longer term expansion in tissue culture plastic (TCP) well‐plates (described in Figure 2a, see Supporting Information for methods). Eleven chemistries (mixed 2:1 v/v) taken forward for scale‐up experiments, included high attachment (D:O, D:Q, B:L, H:N, D:F, P, and B:O ranked 1–74 by total nuclei count; Table S3, Supporting Information) and low attachment (E:M, B:P, K:A, and L:M ranked below 100 by nuclei count; Table S3, Supporting Information) candidates (Figure S5, Supporting Information). In this 96 well plate format, four chemistries failed to support initial attachment at 24 h: D:O, B:O, L:M, and K:A, likely due to the dissolvability of polymers with isopropyl alcohol solvent during coating (see production of polymer coated 6‐well plates methods, Supporting Information), which was selected for its compatibility on TCP. Four further chemistries failed to maintain attachment at 72 h: P, H:N, B:P, and E:M despite showing attachment at 24 h. (Figure S6, Supporting Information). Whilst three chemistries (D:Q, D:F, and B:L) were able to support hPSC attachment and OCT4 pluripotency at 24 (Figure 2b–d) and 72 h (Figure 2e–f) time points at scale‐up were also high attachment candidates (from co‐polymer array screen). From the three chemistries, co‐polymer D:Q was able to support high attachment of hPSCs that was most comparable with Matrigel controls, and was thus selected for long‐term scale‐up experiments.

Co‐polymer D:Q, which comprises of TCDMDA mixed with butyl acrylate (BA) (2:1 v/v) was applied to 6‐well TCP plates of ≈10 cm^2^ base surface area per well (see Supporting Information for methods). Surface characterization by TOFSIMS after scaled production confirmed characteristic peaks of TCDMDA (C_5_H_7_^+^ m/z = 67.05) and BA (C_4_H_9_^+^ m/z = 57.07) (Figure S7, Supporting Information). In addition, atomic force microscopy revealed nanoscale topography in both deformation and modulus images (**Figure** **3**a,b). This indicated that nanoscale phase separation of the monomers occurred before polymerization resulting in the equivalent of a blend of polyBA (minor component, 30% v/v as ≈50 nm islands) in a continuous phase of polyTCDMDA (major component, 70% v/v), rather than an uniform surface that would be representative of a co‐polymer. Hence, D:Q will be referred to here as poly(TCDMDA‐blend‐BA).

**Figure 3:**
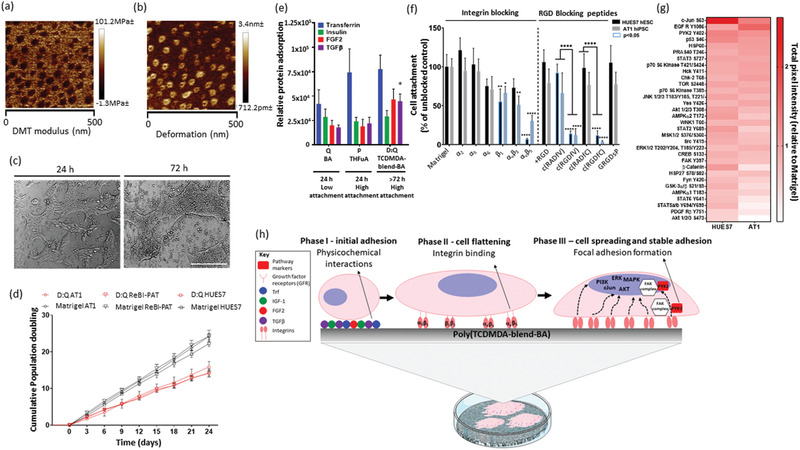
Characterization of poly(TCDMDA‐blend‐BA) surface. Atomic force microscopy a) Derjaguin‐Muller‐Toporov (DMT) modulus and b) deformation micrographs of poly(TCDMDA‐blend‐BA) surface coated on poly(styrene) six well plates showing a nanoscale blend of polyBA (≈50nm islands of minor component, 30% v/v) in polyTCDMDA (background, major component, 70% v/v). c) Representative brightfield images of ReBl‐PAT attachment and maintenance at 24 and 72 h of culture. d) Growth curves of hESC: HUES7 and hiPSC: AT1 and ReBl‐PAT lines on poly(TCDMDA‐blend‐BA) and Matrigel presented as cumulative doubling time ([duration of culture x log2] / [log10 (final cell concentration/initial cell concentration)]). e) LESA‐MS/MS quantification of adsorbed proteins: FGF2, TGF*β*1, insulin, and transferrin from E8 medium on polymer surface after 1 h of incubation (*N* = 3). Bar graph represents ±SEM. One‐way Anova statistical tests were performed and compared between chemistries for each protein (*p < 0.05). f) Blocking of integrins and RGD‐blocking peptides (see Table S4, Supporting Information for details) for 24 h on poly(TCDMDA‐blend‐BA) in E8. Bar graphs presented as mean and error bars represent ±STDEV. (*N* = 3, *n* = 3). One way ANOVA followed by Tukey's multiple comparison tests (*p < 0.05, ^**^ *p* < 0.01, ^****^ *p* < 0.0001) were performed. Statistical differences denoted with black asterisks represent comparisons between polymers and Matrigel unless indicated otherwise. g) Phosphokinase array blots were quantified using Image Studio Software for HUES7 (*N* = 2, *n* = 4) and AT1 (*N* = 1, *n* = 2). Heatmap represents total intensity values per phosphorylated kinase normalized to background intensity and HSP60 internal control. Graph shows Mean + STDEV. h) Schematic to summarize identified hPSC and poly(TCDMDA‐blend‐BA) interactions. The upper panel is zoomed in single cell‐polymer interactions from lower image showing hPSC colonies attached on a well of cultureware coated with poly(TCDMDA‐blend‐BA). In brief (left to right), adsorption of E8 proteins mediate initial phase of cell attachment (phase I) followed by integrin engagement (phase II) which subsequently promote key hPSC signaling pathways (phase III).

To evaluate whether poly(TCDMDA‐blend‐BA) could support hPSC attachment, proliferation, and expansion across serial passages, we evaluated the performance of ReBl‐PAT, and two other lines (AT1,^[^ ^31^ ^]^ an in‐house hiPSC line; HUES‐7,^[^ ^34^ ^]^ a human embryonic stem cell (hESC) line from Harvard University). At scale‐up, we demonstrated that all three hPSC lines cultured on poly(TCDMDA‐blend‐BA) could readily attach and proliferate, reaching confluency every 72 h across 8 serial passages. hPSCs showed a similar morphology to those growing on Matrigel (serving as a positive control) (Figure 3c). Whilst population doubling time on poly(TCDMDA‐blend‐BA) (Figure 3d; AT1: 40 h ± 16 h, HUES7: 40 h ± 13 h) was slower than Matrigel (AT1: 24 h ± 7 h, HUES7: 26 h ± 5 h) and laminin 521 (≈24 h) it was comparable to commonly used ECM vitronectin (36 h) and previously reported polymers for hPSC culture.^[^ ^29^, ^35^ ^]^ The hPSC lines also retained stable karyotypes (46, XY for HUES7; 46, XY for ReBl‐PAT; 46, XX for AT1; Figure S8 and methods Supporting Information) and maintained pluripotent marker expression of OCT4, NANOG, SOX2, TRA181, and SSEA4 confirmed by flow cytometry (Figure S9a, Supporting Information), quantitative real‐time PCR (Figure S9b, Supporting Information) and immunostaining (Figure S9c, Supporting Information, >80%) after 5 serial passages.

We and others have shown that protein adsorption to polymer substrates from culture media is a critical step for mediating initial cell attachment.^[^ ^21^, ^36^, ^37^ ^]^ We therefore next evaluated adsorption of proteins from E8 culture medium on poly(TCDMDA‐blend‐BA) by liquid extraction surface analysis‐tandem mass spectrometry (LESA‐MS/MS).^[^ ^38^ ^]^ Adsorption of medium‐derived proteins to the polymer interface was assessed in the absence of cells for an optimized time of 1 h.^[^ ^27^, ^39^, ^40^, ^41^ ^]^ Followed by a rinsing procedure designed to retain species bound to the surface for analysis by surface sampling (Figure 3e, see Supporting Information for methods). Whilst minimal in its formulation, E8 includes key pluripotency‐maintaining factors: basic fibroblast growth factor (FGF2) and transforming growth factor beta 1 (TGFβ1); as well as insulin and transferrin, which are required for hPSC attachment and survival.^[^ ^1^ ^]^

The amount of each protein adsorbed by the polymer poly(TCDMDA‐blend‐BA) capable of supporting attachment for >72 h was compared to low and high attachment polymers from the short‐term 24 h attachment experiments. These are denoted as monomers Q and P in Figures 1c and 2, polyBA and polyTHFuA, respectively in Figure 3e. Of the four proteins detected, significantly higher TGF*β*1 adsorption was observed on poly(TCDMDA‐blend‐BA) (*p* < 0.05) compared to polymers which only supported attachment for 24 h. Since TGF*β*1 is an important factor for maintaining hPSC pluripotency,^[^ ^1^ ^]^ it is likely that TGF*β* adsorption contributes to the success of poly(TCDMDA‐blend‐BA) in long‐term maintenance of hPSCs in their undifferentiated state.

By 24 h, active cell adhesion was evident by cells altering their morphology from round to flattened and spread‐out cell body by re‐organization of the cytoskeleton driven by integrin‐mediated attachment, which subsequently promoted key hPSC migration and proliferation signaling pathways.^[^ ^42^, ^43^ ^]^ At this time point, we explored integrin‐based mechanisms mediating active hPSC attachment on the poly(TCDMDA‐blend‐BA) surface; antibodies and RGD (arginine‐glycine‐aspartate)‐based peptides were used to block integrins known to be highly expressed on the hPSC surface (Figure 3f; see Table S4, Supporting Information for details).^[^ ^44^, ^45^ ^]^ Antibody‐mediated blocking of vitronectin‐binding *α*_v_ *β*_3_ (AT1, *p* < 0.01) and *α*_v_ *β*_5_ (*p* < 0.0001) integrins, the Matrigel ‐binding *β*_1_ integrin, as well as RGD‐peptides binding *α*_v_ *β*_3_ c(RGDfV), (*p* < 0.0001) and *α*_v_ *β*_5_ c(RGDfC) (*p* < 0.0001), significantly attenuated hPSC attachment to poly(TCDMDA‐blend‐BA).^[^ ^46^, ^47^, ^48^, ^49^ ^]^ Expression of key integrins (*α*_v_, *α*_5_, *β*_1_, *β*_4_, and *β*_5_) was also confirmed through western blotting (Figure S10a,b, Supporting Information). Integrin‐mediated hPSC attachment to synthetic polymer substrates including poly(HPhMA‐co‐HEMA), notably of *β*_1_ and *α*_v_ integrins, has been previously reported.^[^ ^17^, ^21^ ^]^ These data suggest that *α*_v_ *β*_3_ and *α*_v_ *β*_5_ heterodimers play an important role for mediating hPSC attachment to poly(TCDMDA‐blend‐BA), however, integrins are likely to interact in a complex manner and therefore can form a number of different homo‐ or hetero‐ dimers. We can also infer that the identified integrins which were mainly vitronectin binding are likely to influence subsequent hPSC signaling when the cell cytoskeleton is re‐organized for stable cell adhesion for growth expansion.

Intracellular signaling profiling of hPSCs cultured on poly(TCDMDA‐blend‐BA) confirmed maintenance of stem cell integrity. We now wished to explore the impact of these interactions on molecular signaling pathways. A phospho‐kinase array kit (R&D) containing a panel of critical markers was used to assess intracellular signaling events in two independent hPSC lines (AT‐1, HUES7) (Figure 3g).

Expression profiles for several kinases appeared to be cell line specific. However kinases: c‐Jun (S63), EGFR (epidermal growth factor receptor, Y1086) and PYK2 (tyrosine kinase encoded by PTK2B, Y402) were differentially phosphorylated on poly(TCDMDA‐blend‐BA) compared to Matrigel in both hPSC lines (Figure 3g). These kinases activate important downstream hPSC proliferation pathways (including phosphoinositide 3‐kinase/AKT, c‐Jun, extracellular‐signal‐regulated kinase/mitogen‐activated protein kinases), most likely initiated by integrin engagement (including *α*_v_, *β*_1_, and *β*_3_) based on the integrin‐interface characterization experiments on poly(TCDMDA‐blend‐BA) (Figure 3c; Figure S10, Supporting Information).^[^ ^50^, ^51^, ^52^ ^]^ Alternatively, adsorbed E8 factors, all four of which were confirmed for poly(TCDMDA‐blend‐BA), can promote hPSC signaling pathways by activating important growth factor receptors (e.g. insulin‐like growth factor receptor, EGFR, fibroblast growth factor receptor, and transferrin receptor) for hPSC signaling (proposed mechanism summarized in Figure 3h).

In addition to maintaining hPSC attachment and pluripotent signaling, the value of these cells for maintaining their differentiation capacity to multiple lineages is of significant use in biomedical and regenerative medicine applications. Therefore, after serial passaging on poly(TCDMDA‐blend‐BA), directed differentiation of hPSCs toward the three germ layers was performed.^[^ ^11^ ^]^

Definitive endoderm SOX17 and FOXA2 positive cells were achieved after two days of WNT pathway activation with the GSK‐3 inhibitor CHIR99021 (Figure S11a, Supporting Information). hPSC differentiation to SOX1 and PAX6 positive neural progenitors of the ectoderm lineage was achieved with modulators of the TGF‐*β* superfamily (dual SMAD inhibitors dorsomorphin‐1 and SB431542) and WNT (XAV939) pathways (Figure S11b, Supporting Information). Functional contractile cardiomyocytes (mesoderm) cells were formed using modulators of the TGF‐*β* (activin A and BMP4) and WNT (KY02111 and XAV393) pathways for 8–12 days (Figure S11c, Supporting Information). Thus, achieving multi‐lineage differentiation, confirmed that stem cell integrity was retained after serial passaging on poly(TCDMDA‐blend‐BA).

In summary, a high throughput combinatorial materials discovery approach identified poly(TCDMDA‐blend‐BA) for long‐term hPSC culture in commercial defined E8 medium, without the need for addition of xenogenic factors or attachment‐mediating proteins from culture medium. This simplified system has advanced our understanding of the factors at the bio‐interface between media and polymer controlling stem cell response. Protein adsorption of chemically defined E8 medium was compared between different polymers using mass spectrometry methods. Together with cell‐based assays, which investigated cell‐polymer interactions, this enabled us to report on mechanisms for cell adhesion and intracellular signaling which corroborated well with our previous work e.g. poly(HPhMA‐co‐HEMA) and other culture systems used routinely within the field.^[^ ^1^, ^5^, ^6^, ^7^, ^9^, ^17^, ^21^, ^33^, ^39^, ^46^, ^49^ ^]^ This material constitutes a break‐through for the application of hPSCs, addressing current limitations of scalability and production costs which could be reduced by ≈350 fold; costing ≈$0.0003 per well in a 96‐well plate (factoring in a 10% saving from bulk orders based on BA # 234923) compared to ≈$0.1 per well in a 96 well plate for current gold standard substrates including Matrigel (Corning #11573620) and Synthemax (Corning # CLS3535).

## Experimental Section

Complete methodology of polymer synthesis, characterization, and cell‐based assays can be found in the Supporting Information.

## Conflict of Interest

The authors declare no conflict of interest.

## Supporting information

Supporting Information
