# Supplementary material for: Discovery of a Novel Polymer for Xeno‐Free, Long‐Term Culture of Human Pluripotent Stem Cell Expansion
Source: Adv Healthc Mater. 2020 Dec 28;10(6):2001448. doi: 10.1002/adhm.202001448 (PMC11469126; doi:10.1002/adhm.202001448)
Supplement: Supplementary file 1 — Supporting Information [file ADHM-10-2001448-s001.pdf]

WILEY-VCH

© 2020 Wiley-VCH GmbH

ADVANCED HEALTHCARE MATERIALS

Supporting Information

for *Adv. Healthcare Mater.*, DOI: 10.1002/adhm.202001448

Discovery of a Novel Polymer for Xeno-free, Long-term Culture of Human Pluripotent Stem Cell Expansion

Aishah Nasir[#1], *Jordan Thorpe*[#1], *Laurence Burroughs*[#2], *Joris Meurs*[2], *Sara Pijuan-Galito*[1], *Derek J. Irvine*[3], *Morgan R. Alexander*[*2] *and Chris Denning*[*1]

Discovery of a Novel Polymer for Xeno-free, Long-term Culture of Human Pluripotent Stem Cell Expansion

Aishah Nasir[#1], *Jordan Thorpe*[#1], *Laurence Burroughs*[#2], *Joris Meurs*[2], *Sara Pijuan-Galito*[1], *Derek J. Irvine*[3], *Morgan R. Alexander**[2] *and Chris Denning**[1]

[#]Joint first authors

*Joint senior and corresponding authors

Dr. A. Nasir, Dr. J. Thorpe, Dr. L. Burroughs, J. Meurs, Dr. S. Pijuan-Galito, Prof. D. J. Irvine, Prof. M. R. Alexander, Prof. C. Denning.

[1]Division of Cancer & Stem Cells, Biodiscovery Institute, [2]School of Pharmacy, [3]Department of Chemical and Environmental Engineering, University of Nottingham, Nottingham, NG7 2RD, UK

Corresponding author emails: morgan.alexander@nottingham.ac.uk, chris.denning@nottingham.ac.uk

Supporting Information

Routine cell culture

All cell culture experiments were performed in a type II Biological Safety Cabinet, and cells were incubated in a humidified incubator, at 37°C and 5% CO2 (Heracell). Three hPSC lines used in this study, including the hESC line, HUES7 (used between passages 25-35) and the hiPSC cell lines: ReBl- PAT (used between passages 20-30) derived from a skin punch biopsy from a male subject and AT1 (used between passages 20-30) derived from dental pulp of a female subject (as previously described),[1] were routinely maintained on 1:100 Matrigel coating (BD Biosciences, UK) in Essential 8™ medium (E8, LifeTechnologies). In brief, cells were passaged at 70-80% confluency every 3 days by washing once with Ca^{2+}/ Mg^{2+}-free Phosphate Buffer Saline (PBS, Gibco #14190-094), followed by incubation with TrypLE Select (LifeTechnologies) for 2-3 minutes at 37°C, with tapping of flasks to dissociate cells. Afterwards, hPSC were resuspended in E8 supplemented with 10µM Y-27632 (ROCKi, Tocris Bioscience #1254/10) and seeded into new MT-coated flasks at approximately 20000 cells/ cm^2. Medium was changed every day.

Polymer microarray synthesis and preparation

Polymer microarrays were fabricated using methods previously described.[2, 3] Briefly, polymer microarrays were printed onto polyHEMA (4% w/v Sigma, in ethanol (95% v/v in water)) dip coated glass slides using a XYZ3200 dispensing station (Biodot) and quilled metal pins (946MP6B, Arrayit) under an argon atmosphere maintaining O_2 < 2000 ppm, 25°C and 35% humidity. Polymerization solutions consisted of monomer (50% v/v) in dimethylformamide with photoinitiator 2,2-dimethoxy-2-phenyl acetophenone (1% w/v), and were polymerized in-situ using UV light irradiation. Three replicates of 284 monomers were printed per slide for the first generation array (see Figure S1 for structures and Table S1 for monomer list). For the second generation array, the polymerisation solutions consisted of major and minor monomers in a 2:1 (v/v) ratio. Three replicates of 342 co-polymers combinations were printed per slide. Monomers were purchased from Sigma, Scientific Polymers and Polysciences and were used as received. Top and bottom array surfaces were sterilised with UV light for 15 minutes and washed with sterile Ca^{2+}/Mg^{2+} -free Phosphate Buffer Saline (PBS, Gibco) before culturing with hPSCs.

Microarray screening and data acquisition

0.75×10^6 REBl-PAT cells were seeded in E8 medium supplemented with 10μM Y-27632 (ROCKi, Tocris Bioscience) on each array and incubated at 37°C with 5% CO_2 for 24 h and 48 h timepoints at which point array samples were fixed with 4% paraformaldehyde for quantification. Arrays were immunostained for OCT4 expression and counterstained with 4',6-diamidino-2-phenylindole (DAPI) (see immunostaining methods for full details) before being mounted with Vectashield Antifade mounting medium (Vector Laboratories and imaged using automated fluorescence microscopy (IMSTAR). Attachment was analysed in CellProfiler ver. 2.2.0 (Broad Institute).[4] Manual background correction was also applied to images prior to using in-built "identify primary objects" algorithm using a three-class Otsu adaptive thresholding method to identify and quantify nuclei in DAPI and OCT4 channels with manual check for quality control. Assessment of co-polymer

combinations for second generation screen can be readily performed using a synergy ratio (SR). Taking the response of major (y1) and minor (y2) monomers alone, normalised to the fraction (m) present in the co-polymer (y12), the SR can be calculated using the equation: $SR = \frac{y_{12}}{(m \times y_1)+(m \times y_2)}$

A synergistic combination, SR>1, indicates that the cell response for the co-polymer is greater than the response of the individual monomers. Whilst an additive/counteractive combination, SR<1, indicates that the cell response for the co-polymers is less than the response of the individual monomers.

hPSC assessment of polymer candidates coated on 96-well plates

ReBl-PAT hPSCs were seeded at 4.5×10^4 cells/cm^2 on co-polymers selected for scale-up in E8 medium supplemented with Y-27632 where each co-polymer was tested in triplicate wells. Matrigel controls were also included for comparison. Images of five separate fields were obtained per well (n=3 independent repeat) using the Operetta high-content imaging system (Perkin Elmer). Images were analysed using Harmony high-content image analysis software (Perkin Elmer) developed with PhenoLOGIC machine learning algorithms to quantify percentage cell coverage (relative to total areas imaged per well) and mean area of colonies (total cell coverage/no. of colonies). Adhered cells at 72 h were fixed in 4% paraformaldehyde and immunostained for OCT4 and fluorescence microscopy using the Operetta and Harmony was used to quantify total and OCT4+ nuclei (5 fields/well).

Production of polymer coated 6-well plates

Monomers for polymerisation, consisting of individual monomers or 2 monomers mixed at 2:1 (v/v), were mixed in a 9:1 (v:v) ratio with a 10 wt % solution of photoinitiator 2,2-dimethoxy-2-phenyl acetophenone in isopropyl alcohol and coated onto oxygen plasma treated (p_i=0.09 mbar, 100 W, 13.56 MHz RF generator for 10 minutes) tissue culture plastic well-plates. These were then polymerised by exposure to UV light (365 nm, 2 x 15 W, 10 cm distance) for 1 h in an argon glovebox

(<2000 ppm O_2). After polymerization, well-plates were washed three times with isopropanol to remove unreacted polymer, and soaked in dH_2O for 48 h at 37°C. Well-plates were subsequently sterilized with 70% IMS and washed three times with sterile PBS before use.

Surface chemistry analysis

The surface chemistry of array slides and 6-well plates was assessed using time-of flight secondary ion mass spectrometry (ToF-SIMS) and atomic force microscopy (AFM).

ToFSIMS

Measurements were taken using a TOF-SIMS 4 (IONTOF GmbH) instrument using a 25kV $Bi3^{++}$ primary ion source with a pulsed target current of ~1pA and analysed using SurfaceLab 6, IONTOF as previously described.[2]

AFM

Hydrated AFM measurements were acquired using a Bruker Dimension FastScan in PeakForce™ mode using SCANASYST-FLUID+ probes. Samples assessed for surface analysis were incubated in ultrapure MilliQ water (18.2 Ohm) and the probes were calibrated using a 2.6 GPa Bruker polystyrene film sample.[2]

Protein adsorption analysis

Sterilized and washed polymer coated 6 well-plates were incubated in E8 medium supplemented with 10µM Y-27632 dihydrochloride for 1 h at 37°C. Plates were washed with dH_2O (18.2 MΩ, ElgaPure LabWater). Proteins were digested in-situ using microwave-assisted techniques using 0.05 µg/µL trypsin (sequencing grade; Promega, UK) in 100mM ammonium bicarbonate (BioUltra,≥99.5%, Sigma-Aldrich) adapted from previously described methods.[5] Standard methods were used to extract proteins using an extraction solution consisting of acetonitrile (CHROMASOLV®, Riedel-de Haen) and 200 mM ammonium acetate (≥99.0%; Sigma-Aldrich, Gillingham, UK) (1:9 v/v) in LC-MS grade water (CHROMASOLV®, Riedel-de Haen). Samples were analysed by liquid extraction

surface analysis-mass spectrometry (LESA-MS) and introduced to a TriVersa Nanomate (Advion Biosciences, Ithaca, NY) coupled to a Q Exactive plus mass spectrometer (Thermo, San Jose, CA) via nanoelectrospray ionisation (ESI Chip™, Advion Biosciences) using 1.6 kV voltage and 0.6 psi gas pressure (N_2).

hPSC serial passaging on polymer coated 6-well plates

hPSCs were seeded at $7x10^4$ cells/cm^2 in E8 medium supplemented with 10µM Y-27632 dihydrochloride for the initial 24 h of culture. Medium was exchanged every 24 h until cells reached 70-80% confluency at 72 h when cells were fixed or passaged by dissociating with TryPLE select (as described above). hPSCs growth was assessed using an automated cell-viability counter (CEDEX Hi Res Analyser) at each passage (every 72 h). Doubling time (www.atcc.org; [duration of culture x $\log_2$] / [$\log_{10}$ (final cell concentration/seeding concentration)] was calculated for hPSCs and was plotted cumulatively. After 5 serial passages HPSC were karyotyped as previously described.[1]

Flow cytometry

hPSCs serially passaged on polymer substrate (≥ 3 passages) were dissociated into single-cell suspension and fixed with 4% paraformaldehyde. Samples were permeabilized with 0.1% Tween-20 in PBS for intracellular markers and incubated with primary antibodies NANOG (1:100, APCH7 conjugated, BD Biosciences, 560109), SOX2 (1:20, Alexa Fluor 647-conjugated, R&D Systems, IC2018R), TRA181 (1:100, PE-conjugated, Invitrogen, 12-8883-82) and SSEA4 (1:20, fluorescein-conjugated, R&D Systems, FAB1435F) diluted in PBS for 1hr at RT. The FC500 flow cytometer (Beckman Coulter) was used to acquire measurements and expression was quantified with Kaluza analysis software (Beckman Coulter).

Attachment blocking

hPSCs were harvested and re-seeded in E8 medium with the addition of integrin blocking antibodies (10µg/ml for each antibody) or RGD-blocking peptides (15µg/ml) for 24 h (see table S2). Cells were washed three times with PBS, fixed with 4% paraformaldehyde and counterstained with DAPI. Fluorescence images acquired using the Operetta (Perkin Elmer) were quantified for total nuclei count per condition in Harmony image analysis software (Perkin Elmer).

Integrin expression by Western Blot

hPSCs serially passaged on polymer (≥ 3 passages) were lysed using RIPA buffer (Cell Signalling Technologies #9806) supplemented with PMSF (Phenylmethylsulfonyl fluoride, Sigma 10837091001). Total lysate protein was determined using Pierce BCA Protein Assay Kit (Thermo Fisher Scientific # 23225) following manufacturer's instructions. LDS NuPAGE Sample Buffer (4X) with 2.5% 2-mercaptoethanol was added to 30µg of protein lysate and run on NuPAGE NOVEX Bis-Tris Gels with MOPS SDS Running Buffer (Thermo Fisher Scientific #NP0008, #NP0001). Samples were transferred to an Amersham Protran 0.45m nitrocellulose blotting membrane (GE Healthcare Life Science #10600124). Membranes were incubated with following antibodies α_5 (#4705), α_v (#4711), β_1 (#9699), β_4 (#14803) and β_5 (#3629)integrins (all purchased from Cell Signalling Technology and diluted 1:500), Nanog (clone 7F7.1, Millipore, MABD24, 1:500) and β-actin (Millipore, MA1140, 1:2000). Membranes were developed using West Pico PLUS Chemiluminescent Substrate (Thermo Fisher Scientific #34577) on an LAS-400 Imaging system.

Proteome Profiler Array

Human Phospho-Kinase Array (R&D systems, ARY003B) was performed according to manufacturer's instructions (www.rndsystems.com) on hPSCs serially passaged on polymer and Matrigel^{TM} in parallel (≥ 3 passages). Array blots were imaged using ImageQuant LAS-4000 (Fujitsu

Life Sciences) and analysed using Image Studio Software (LI-COR, version 5.2.5) where individual total signal intensity was measured by manual gating. All intensity values were normalized to background intensity and HSP60 internal control according to manufacturer's instructions. Changes were quantified by comparison between Matrigel^{TM} and polymer conditions.

Tri-lineage differentiation

hPSCs serially passaged (≥ 3 passages) were harvested and seeded at $2x10^4$ – $1x10^5$ cell/cm^2 and expanded in E8 medium for 2 days with daily media exchanges. All directed differentiation protocols were performed on hPSCs at day 2. For definitive endoderm differentiation, media was replaced by RPMI supplemented with B27 without insulin (LifeTechnologies 0080085-SA) and CHIR99021 (2µM; STEMCELL Technologies, 72052) for a further 2 days with daily media exchanges. To produce neural progenitors of the ectoderm lineage, media was replaced by Advanced DMEM/F-12 (LifeTechnologies) supplemented with 1% L-glutamine (Life Technologies), 1% CD Lipid Concentrate (Life Technologies) 7.5µg/ml Transferrin (Sigma-Aldrich), 14µg/ml Insulin (Sigma Aldrich), 0.1mM β-mercapto-ethanol, 10µM SB431542 (Tocris) and 1µM Dorsomorphin-1 (Tocris) and 2µM XAV939 (STEMCELL Technologies) for 5 days with daily media exchanges. Differentiation to cardiomyocytes was achieved using methods previously described.[1]

Immunostaining

Adherent cells were fixed in 4% paraformaldehyde (Sigma-Aldrich, UK) at room temperature (RT) for 20 minutes and permeabilized with 0.1% Triton-X100 (Sigma-Aldrich, UK) in PBS at RT for 15 minutes. Non-specific binding was blocked with 4% serum (Sigma-Aldrich, UK) in PBS at RT for 1 h. Samples were incubated overnight at 4°C with primary antibodies OCT4 (1:200, Santa Cruz Biotechnology, SC-5279), TRA181 (1:200, Millipore, MAB4381), SSEA4 (1:100, Millipore), FOXA2 (1:500, Sigma-Aldrich 07-633), SOX17 (1:100, R&D AF1924), SOX1 (1:100, R&D AF3369), PAX6 (1:100, R&D AF8150) and cardiac α-actinin (1:800, Sigma-Aldrich A7811) diluted in blocking solution with the addition of 0.1% Triton X-100 for nuclear stains. Samples were washed

with 0.1% Tween-20 (Sigma-Aldrich, UK) and incubated with Alexa Fluor secondary antibodies (Life Technologies) 1:400 in blocking solution for 1 h at RT in the dark. Cells were washed with 0.1% Tween-20 and nuclei were counterstained with 0.5µg/ml DAPI (4',6-diamidino-2-phenylindole, Sigma-Aldrich D9542).

RNA Extraction, cDNA Synthesis and qPCR

Total RNA was extracted from cell pellets using the NucleoSpin® RNA kit (Machery-Nagel), and reverse transcribed using SuperScript™ III Reverse Transcriptase kit (ThermoFisher), according to manufacturer's instructions.

Real-time qPCR experiments were performed with GoTaq® qPCR Gene Expression assays (Promega) following manufacturer's instructions. Briefly, GoTaq® mastermix (A6001) including the primers of interest (hOCT4 (Forward Primer (FP): GCTCGAGAAGGATGTGGTCC , Reverse Primer (RP): CGTTGTGCATAGTCGCTGCT), hNANOG (FP: GCAGAAGGCCTCAGCACCTA, RP: AGGTTCCCAGTCGGGTTCA) and hSOX2 (FP: CACTGCCCCTCTCACACATG, RP: TCCCATTTCCCTCGTTTTTCT)) was added to a MicroAmp Fast 96 well plate (#4346907). Subsequently, DNA samples (from initial 500 ng of reverse-transcribed RNA) were added to the plate which was thereafter sealed with a film (#4360954). Amplification was performed in ABI 7500 Real-Time PCR system (Applied Biosystems). Normalisation was performed using the house keeping gene hHPRT (FP: TGACACTGGCAAAACAATGCA, RP: GGTCCTTTTCACCAGCACGCT) and the ΔΔCT method was applied for quantification. [6]

Statistical tests

Experiments were performed in at least three independent experiments unless otherwise stated. Statistical tests (as stated in text) were performed using GraphPad Prism (version 8.1.2, San Diego CA). Statistical outliers were identified and excluded using the robust regression and outlier remover (ROUT) analysis with Q=1%. [7] Heatmaps were plotted using the heatmap.2 function from the gplots

package version 3.1.0.2 in combination with the RColorBrewer package version 1.1-2. Clustering and dendrograms for heatmaps were produced using the complete method with Euclidean distance measure.[8] Assay suitability of co-polymer screen was determined by applying Z-factor statistical calculation:[9]

Z-factor = $1 - \frac{3(\sigma p+\sigma n)}{\mu p-\mu n}$ where μ represents the mean value, σ represents the standard deviation value. Matrigel was used as the positive (p) sample whilst HEMA (background chemistry) was used as the negative (n) control.

References

1. D. Mosqueira, I. Mannhardt, J. R. Bhagwan, K. Lis-Slimak, P. Katili, E. Scott, M. Hassan, M. Prondzynski, S. C. Harmer, A. Tinker, G. W. Smith, L. Carrier, P. M. Williams, D. Gaffney, T. Eschenhagen, A. Hansen, C. Denning, *Human Gene Therapy*. **2018**. *29*, 12 A93-A94.
2. A. D. Celiz, J. G. W. Smith, A. K. Patel, R. Langer, D. G. Anderson, D. A. Barrett, L. E. Young, M. C. Davies, C. Denning, M. R. Alexander, Biomat. Sci. **2014**. *2*, 11, 1604-1611.
3. A. D. Celiz, J. G. Smith, A. K. Patel, A. L. Hook, D. Rajamohan, V. T. George, L. Flatt, M. J. Patel, V. C. Epa, T. Singh, R. Langer, D. G. Anderson, N. D. Allen, D. C. Hay, D. A. Winkler, D. A. Barrett, M. C. Davies, L. E. Young, C. Denning, M. R. Alexander, *Adv Mater*. **2015**. *27*, 27, 4006-12.
4. M.R. Lamprecht, D.M. Sabatini, A.E. Carpenter, *Biotechniques*, **2007**. 42, 1, 71-5.
5. W. Rao, A. D. Celiz, D. J. Scurr, M. R. Alexander, D. A. Barrett*. J Am Soc Mass Spectrom*. **2013**. *24*, 1927-36.
6. T.D. Schmittgen, K.J. Livak, *Nat. Protocols*. **2008**. *3*, 6, 1101-1108.
7. H.J Motulsky, R.E. Brown, *BMC Bioinformatics*. **2006**. *7*, 123.
8. B.S. Everitt, S. Landau, M. Leese, *Cluster Analysis*. Fourth ed. **2001**.
9. J. H. Zhang, T. D. Chung, K.R. Oldenburg, *J. Biomol. Screening*. **1999**. *4*, 2, 67-73.

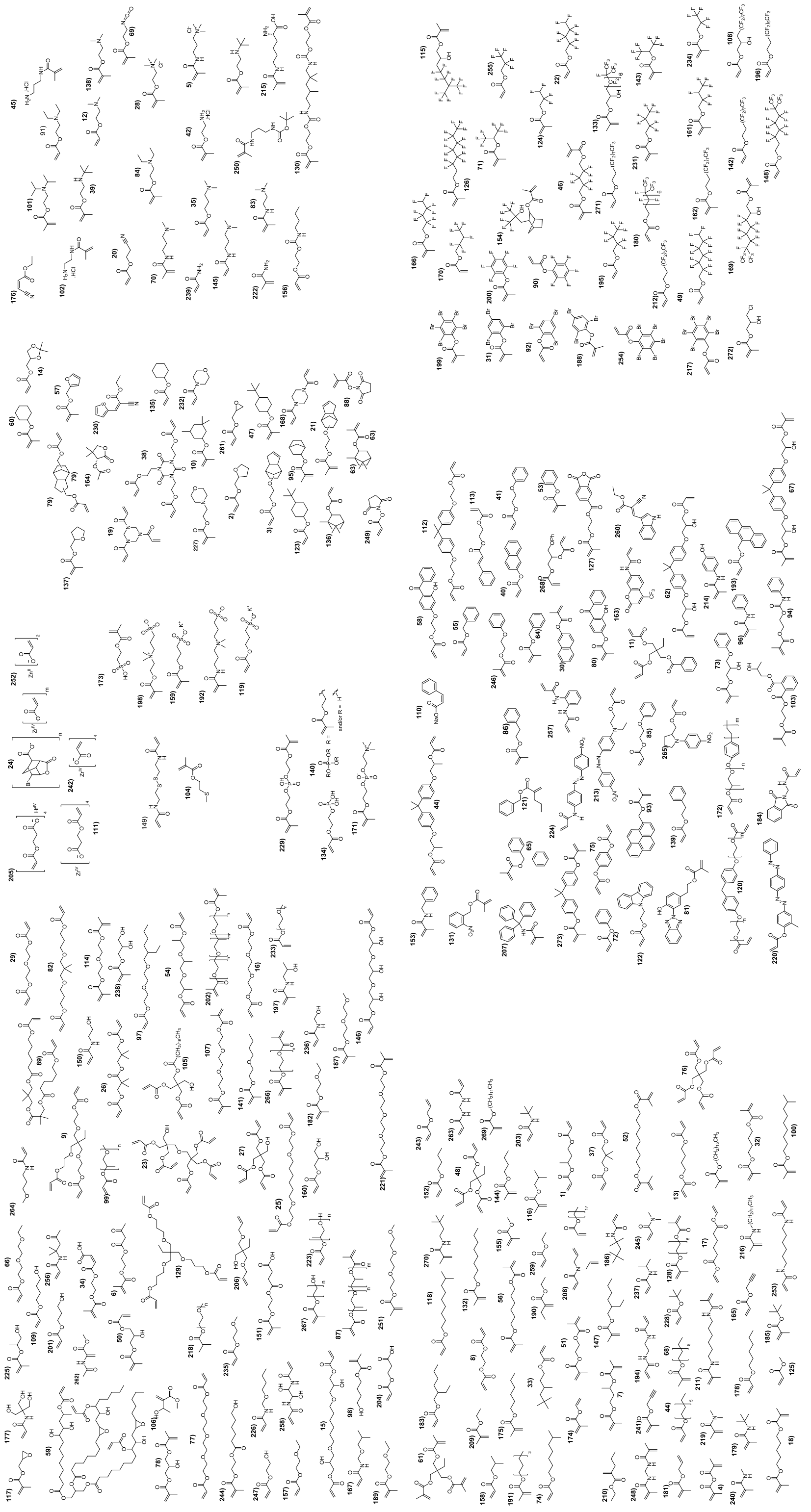

Figure S1: Monomer structures of materials used for the first generation microarray screen. Each structure has been labelled and full IUPAC names are summarised in table S1.

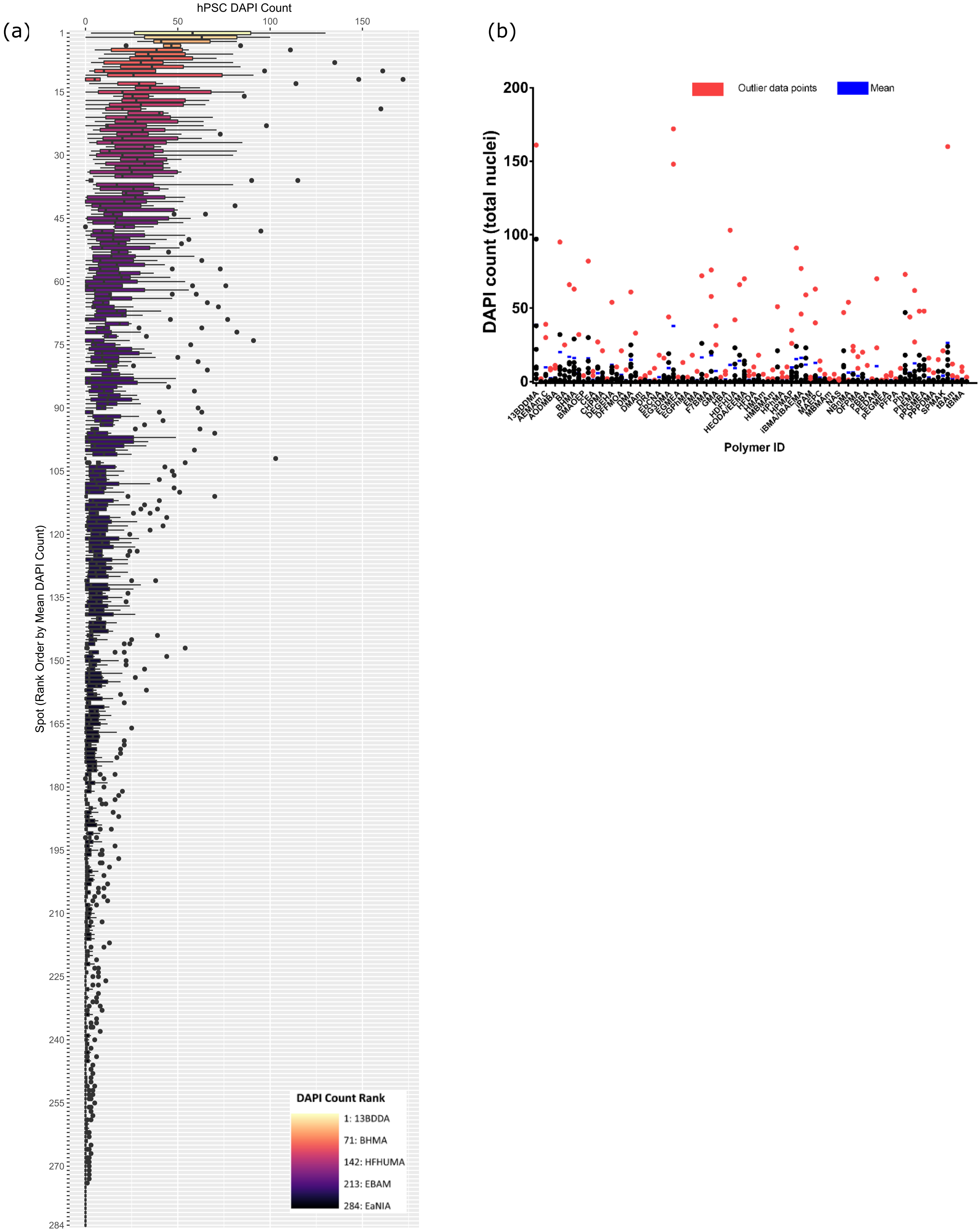
(a)
hPSC DAPI Count
Spot (Rank Order by Mean DAPI Count)
DAPI Count Rank
1: 13BDDA
71: BHMA
142: HFHUMA
213: EBAM
284: EaNIA
(b)
Outlier data points
Mean
DAPI count (total nuclei)
Polymer ID

(c)

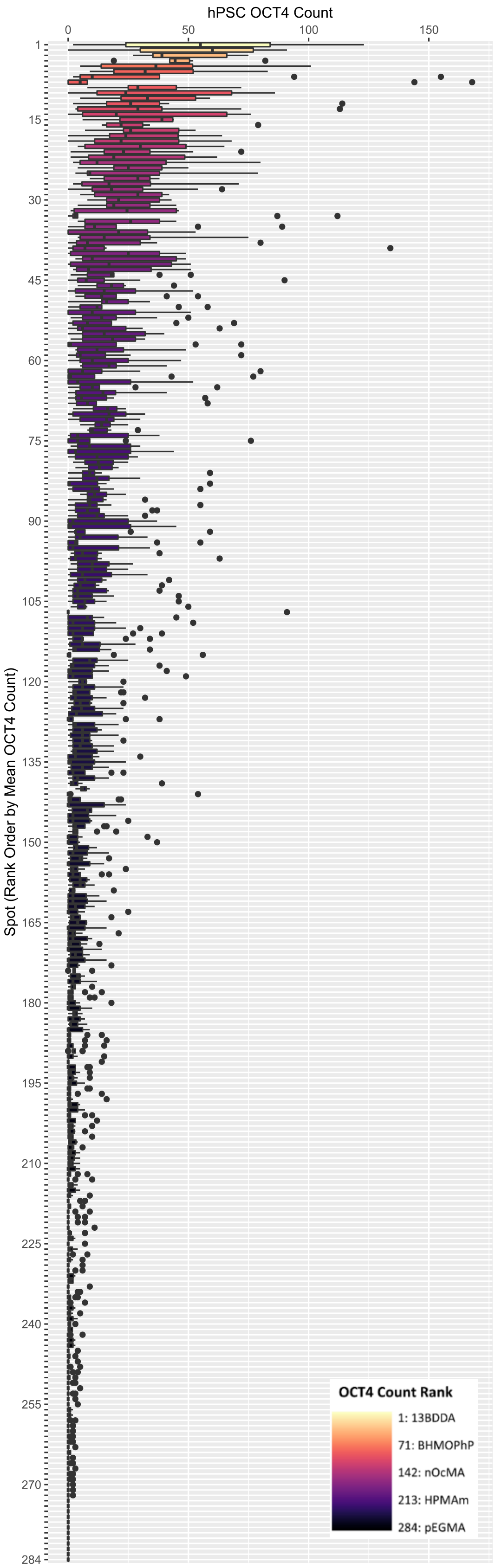

Figure S2 (a) Total cell number (DAPI count) (b) with outlier data points (denoted in red) were calculated using ROUT analysis (Q=1%) c) and OCT4+ count of REBl-PAT hPSCs on 284 monomer microarray ranked high to low (denoted light to dark, see legend) after 24 h. (See Table S2 for rank order 1-284)

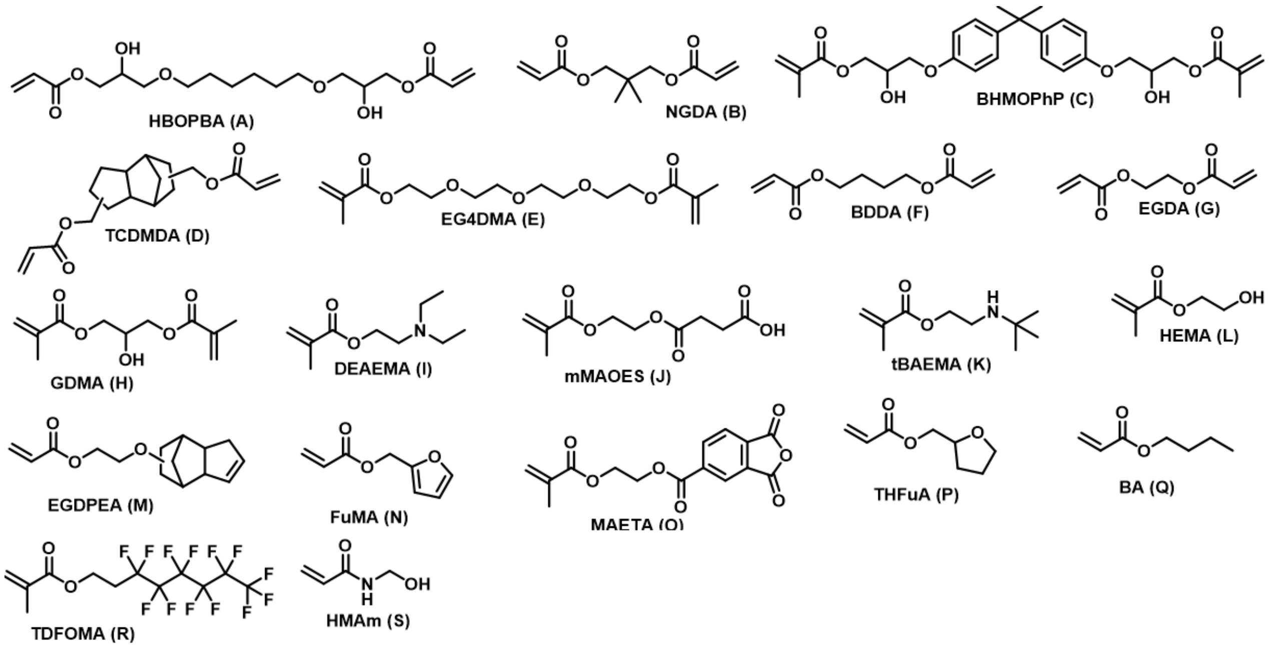

Figure S3: Monomer structures of 19 materials selected for second generation co-polymer screen labelled A-S as referred to in main text.

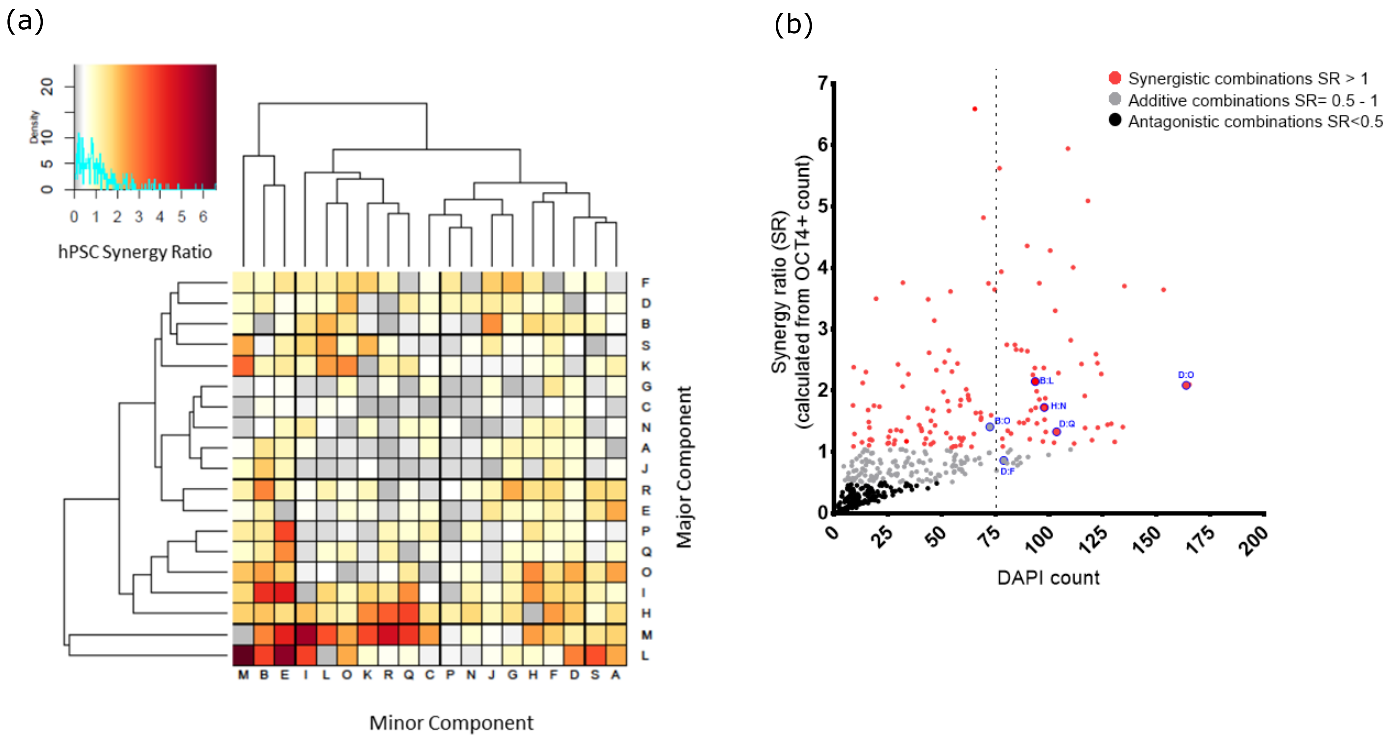

Figure S4: (a) Synergy of co-polymer combinations were quantified as a ratio of OCT4+ attachment for co-polymer to their corresponding homopolymer components (see supplementary information for methods) clustered by Euclidean distance measure. Synergy ratios (SR) >1 are synergistic combinations (denoted yellow - red), SR values = 1 are additive combinations (denoted in white) and SR values <1 are antagonistic combinations (denoted in grey). All letter IDs mentioned are defined in Figure S2. (b) SR scores were plotted against average total cell number (n=9, where n represents the no. of polymer spots). Data has been defined as synergistic (red), additive (grey) or antagonistic. Data points to the right of dotted line represent high attachment polymers. Highlighted data points (blue) are co-polymer candidates selected for scale-up experiments. All attachment data is summarized in table S3.

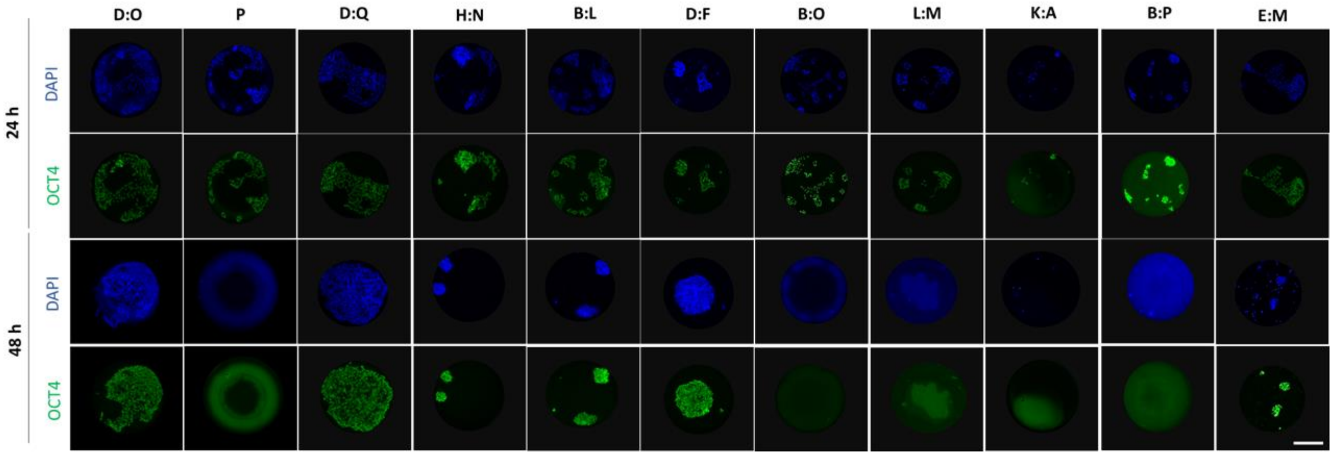

Figure S5: Representative images of OCT4 and DAPI stained REBl-PAT attachment on candidate polymers for scale-up on second generation polymer arrayed slides seeded at 0.75 x10^6 cells/ array at 24 h and 48 h time points in rank order (left to right; quantified from second generation co-polymer array). See Figure S3 for polymer IDs. Scale bar represents 100μm.

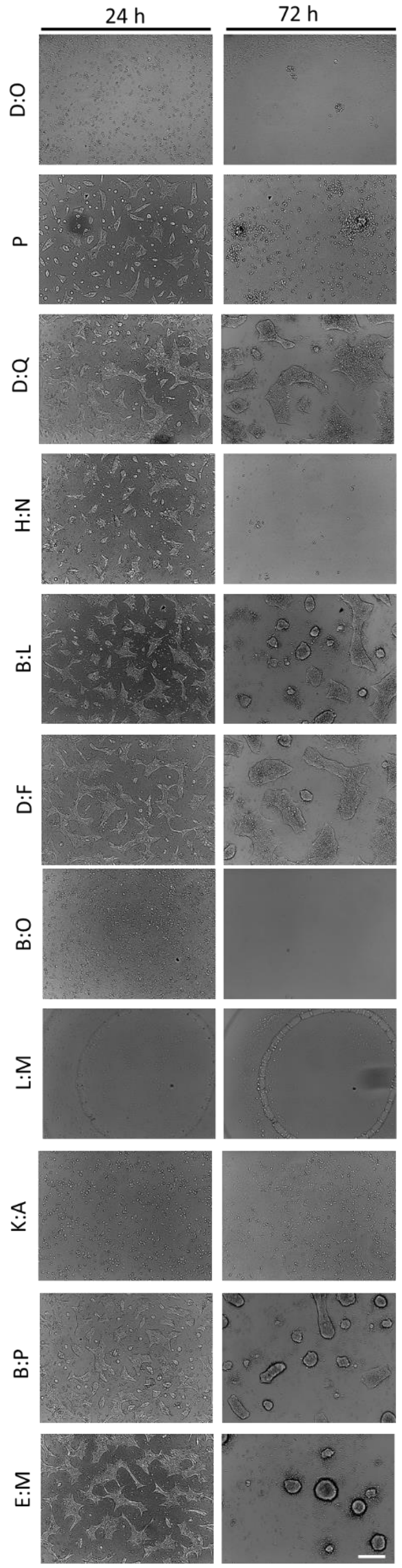

Figure S6: Time-lapse representative brightfield images of REBl-PAT attachment on candidate polymers scaled-up on 96 well plates in rank order (top to bottom) at 24 h and 72 h time-points. Scale bar represents 200µm.

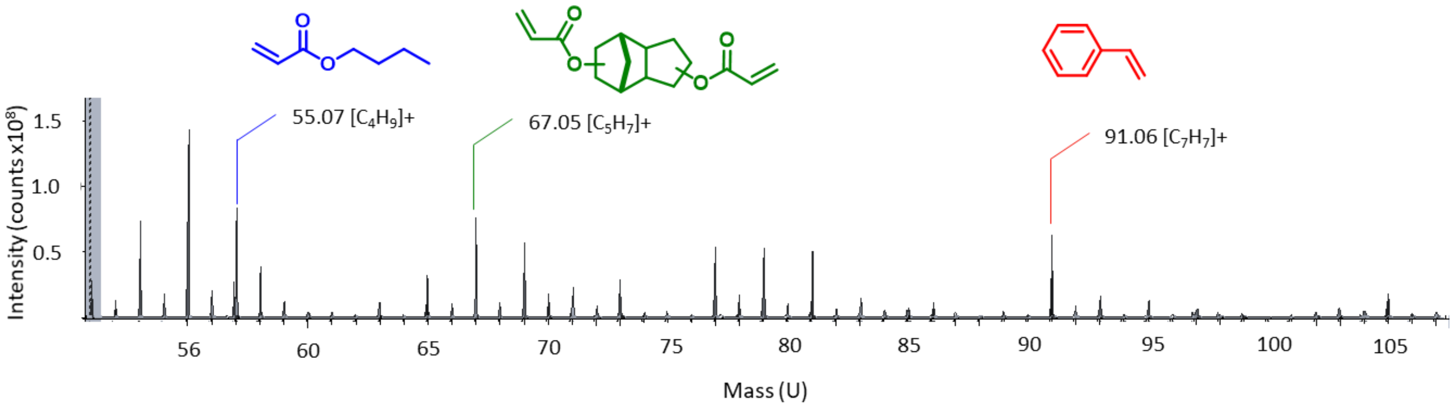

Figure S7: TOFSIMS analysis of poly(TCDMDA-blend-BA) surface on poly(styrene) based tissue culture six well-plates. Ions characteristic of polyBA ($[C_4H_9]^+$ m/z = 57.07, polyTCDMDA ($[C_5H_7]^+$ m/z = 67.05) and poly(styrene) ($[C_2H_7]^+$ m/z = 91.06).(N=3, area analysed =3x3mm, constituent monomers shown for references)

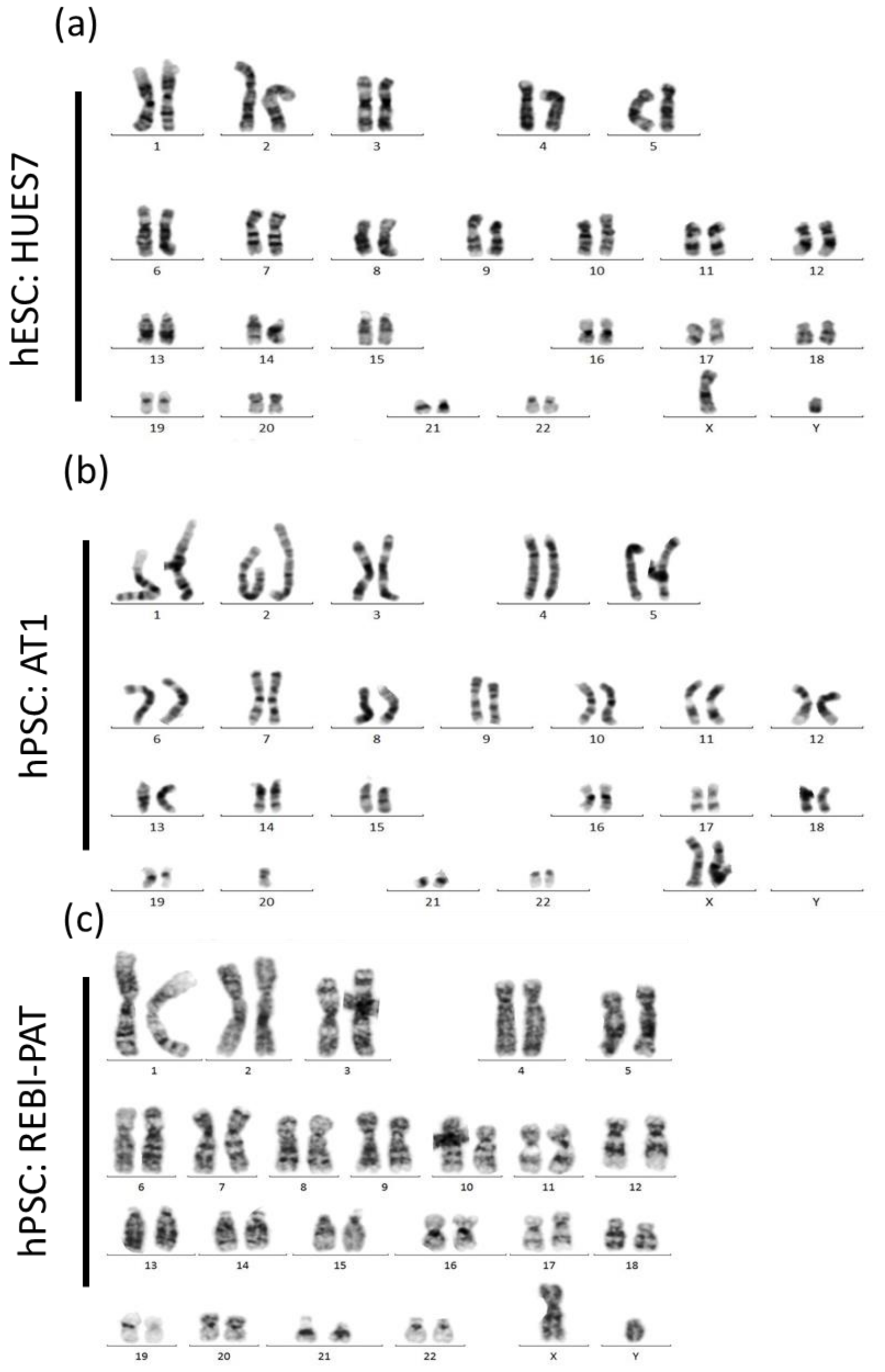

Figure S8: Karyograms observed after 5 serial passages on poly (TCDMDA-blend-BA) for (a) hESC HUES7 (46,XY), (b) hiPSC AT1 (46, XX) and (c) hiPSC REBl-PAT (46, XY) cultured in E8 medium.

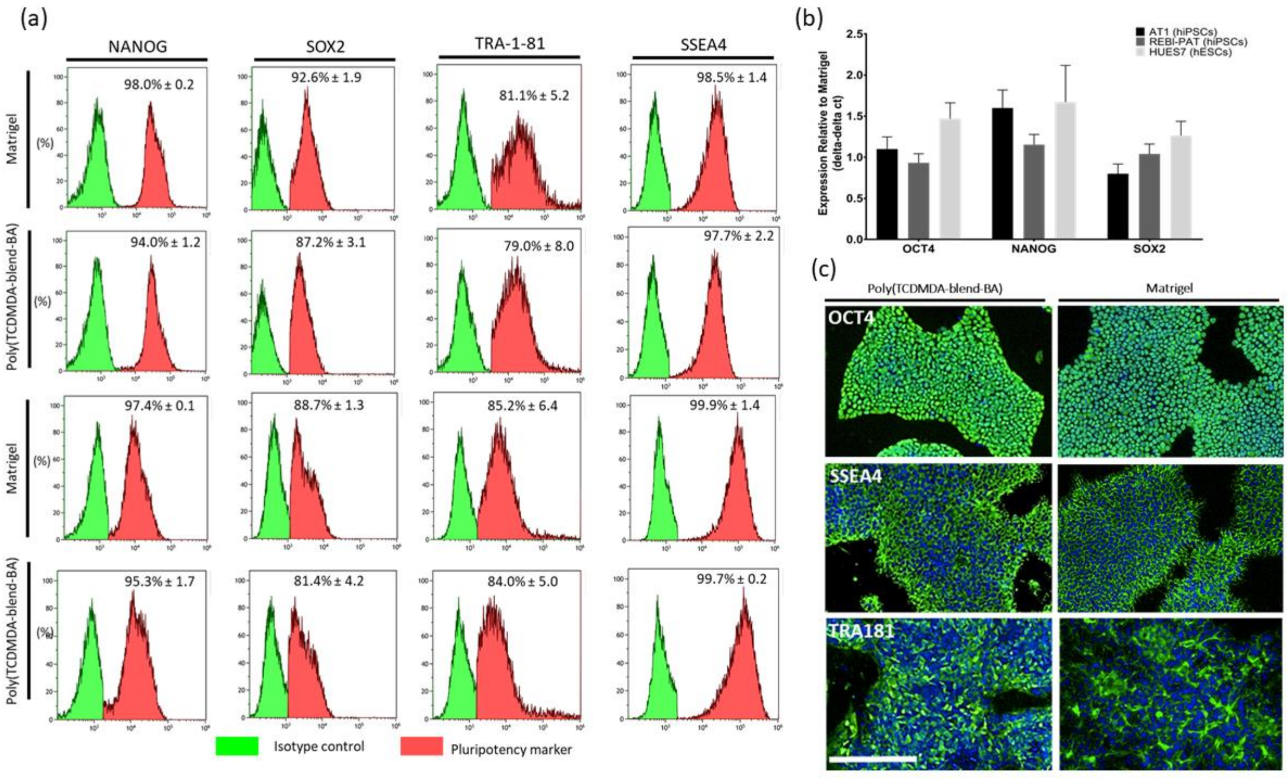

Figure S9: hPSCs (hiPSC AT1 and REBl-PAT lines and hESC HUES7 line) were assessed for pluripotency markers after 18 days (5 serial passages) on poly(TCDMDA-blend-BA) and compared to Matrigel by (a) flow cytometry (b) quantitative real-time PCR, (c) and immunostaining (ReBl-PAT). Scale bar represents 200µm.

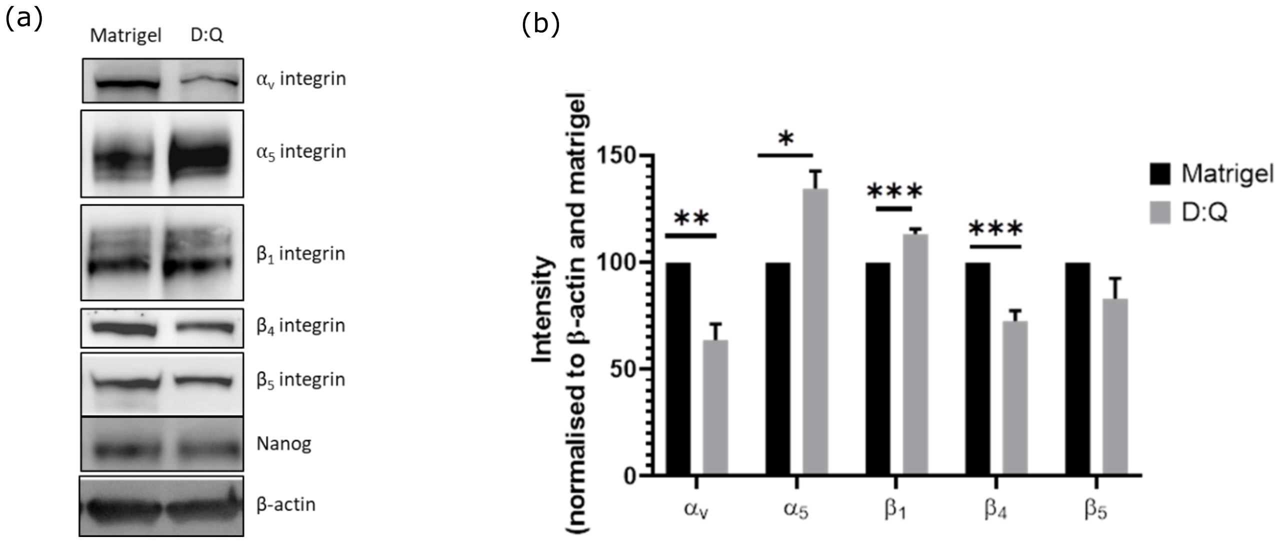

Figure S10: Protein expression of integrin subunits in hiPSC AT1 cells cultured on Matrigel and D:Q (poly(TCDMDA-blend-BA) for at least three serial passages assessed by western blot analysis. (a) Representative images of Western Blotting bands for integrin subunits α_v, α_5, β_1, β_4, β_5; stem cell marker Nanog, and house-keeping protein β-actin, (n=3). (b) Quantification of band intensity for integrin expression in AT-1 hiPSCs ($n \geqslant 3$), bars show Mean ± STDEV; black bars show Matrigel control and grey bars show AT-1 on the hit Polymer. Unpaired t-test were performed, and statistical significance is represented as: *P<0.05, **P<0.01, ***P<0.001.

.

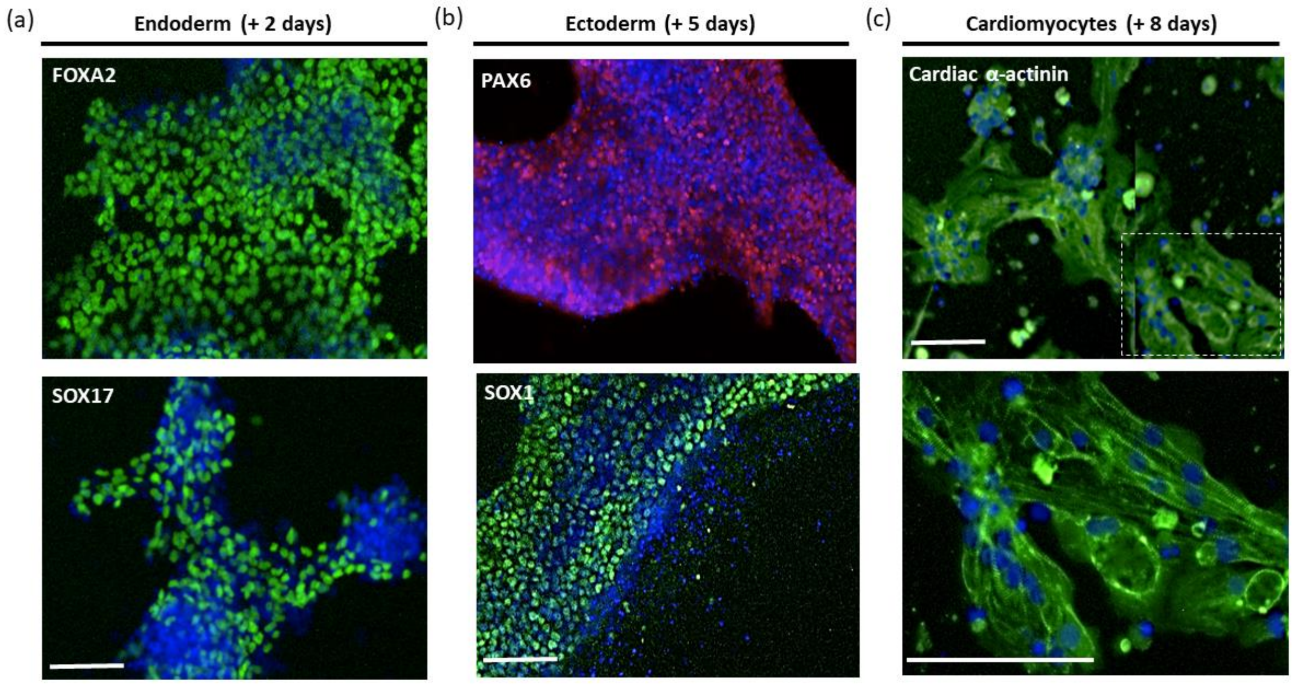

Figure S11: Tri-lineage differentiation of REBl-PAT hPSCs cultured on poly (TCDMDA-blend-BA) for five passages. (a) Definitive endoderm differentiation induced early-stage marker expression of FOXA2 and SOX17 after 2 days. (b) Ectoderm differentiation induced neurogenesis marker expression after 5 days. (c) Mesoderm differentiation induced positive α-actinin expression after 8 days. Scale bars represent 100µm.

Table S1: Full list of monomers included for microarray screens with acronyms and full IUPAC names. Number IDs refer to structures in Figure S1.

No. ID	Acronym	Name
1	13BDDA	Butanediol-1,3 diacrylate
2	THFuA	Tetrahydrofurfuryl acrylate
3	EGDPEA	Ethylene glycol dicyclopentenyl ether acrylate
4	MAAH	Methacrylic anhydride
5	MAPtMA	Methacrylamidopropyltrimethylammonium chloride,
6	MAEA	Methacryloyloxy)ethyl acetoacetate
7	13BDDMA	1,3-Butanediol dimethacrylate
8	EGDA	Ethylene glycol diacrylate
9	TMPETA	Trimethylolpropane ethoxylate triacrylate
10	TMCHMA	Trimethylcyclohexyl methacrylate
11	TMOBDA	Trimethylolpropane benzoate diacrylate
12	DMAEMA	Dimethylamino-ethyl methacrylate
13	BDDA	Butanediol diacrylate
14	SolA	Solketal acrylate
15	HBOPBA	Hexanediylbis[oxy(2-hydroxy-3,1-propanediyl)] bisacrylate
16	E3GDA	Triethylene glycol diacrylate
17	DVAd	Divinyl Adipate
18	PDDMA	1,5-Pentanediol dimethacrylate
19	TAHTA	1,3,5-Triacryloylhexahydro-1,3,5-triazine
20	CNEA	Cyanoethyl acrylate
21	EGDCMA	Ethylene glycol dicyclopentenyl ether methacrylate
22	OFPA	Octafluoropentyl acrylate
23	DPEPHA	Dipentaerythritol penta/hexa-acrylate
24	ZrBNCTA	Zirconium bromonorbornanelactone carboxylate triacrylate
25	HEODA	Hexanediol ethoxylate diacrylate
26	HDMPDA	Hydroxy-2,2-dimethylpropyl 3-hydroxy-2,2-dimethylpropionate diacrylate
27	PETrA	Pentaerythritol triacrylate
28	MAEACl	[2-(Methacryloyloxy)ethyl]trimethylammonium chloride solution
29	DEGDA	Di(ethylene glycol) diacrylate
30	NpMA	Naphthyl methacrylate
31	TBNpMA	Tribromoneopentyl methacrylate
32	14BDDMA	1,4-Butanediol dimethacrylate
33	TMHA	Trimethylhexyl acrylate
34	mMAOEM	mono-2-(Methacryloyloxy)ethyl maleate
35	DMAPA	Dimethylamino-propyl acrylate
36	DDDMA	1,10-Decanediol dimethacrylate
37	NGDA	Neopentyl glycol diacrylate
38	TAIC	Tris[2-(acryloyloxy)ethyl] isocyanurate
39	tBAEMA	Tert-butylamino-ethyl methacrylate
40	NpA	Naphthyl acrylate
41	EGPEA	Ethylene glycol phenyl ether acrylate
42	AEMA.C	2-Aminoethyl methacrylate hydrochloride,
43	LaA	Lauryl acrylate
44	BAPODA	Bisphenol A propoxylate diacrylate
45	APMAm.C	N-(3-Aminopropyl)methacrylamide hydrochloride
46	HFPDA	Hexafluoropent-1,5-diyl diacrylate
47	tBCHA	Tert-butylcyclohexylacrylate
48	TMPTA	Trimethylolpropane triacrylate
49	DFHA	Dodecafluoroheptyl acrylate

50	AOHPMA	Acryloyloxy-2-hydroxypropyl methacrylate
51	EGDMA	Ethylene glycol dimethacrylate
52	NDDMA	1,9-Nonanediol dimethacrylate
53	PhA	Phenyl acrylate
54	TPGDA	Tri(propylene glycol) diacrylate
55	BnA	Benzyl acrylate
56	HDDMA	1,6-Hexanediol dimethacrylate,
57	FuMA	Furfuryl methacrylate
58	BzHPEA	Benzoyl-3-hydroxy-phenoxy)ethyl acrylate
59	ExA	Epoxidized acrylate
60	CHMA	Cyclohexyl methacrylate
61	TMOPTMA	1,1,1-Trimethylolpropane trimethacrylate
62	BPAPGDA	Bisphenol A propoxylate glycerolate diacrylate
63	iBMA	Isobornyl methacrylate:
64	PhMA	Phenyl methacrylate
65	BHMA	Benzhydryl methacrylate
66	DEGEEA	Di(ethylene glycol) ethyl ether acrylate
67	BAGDA	Bisphenol A glycerolate diacrylate
68	SMA	Stearyl methacrylate
69	iCEMA	Isocyanatoethyl methacrylate
70	DMPMAm	N-[3-(Dimethylamino)propyl]methacrylamide
71	HFiPA	Hexafluoroisopropyl acrylate
72	BnMA	Benzyl methacrylate
73	HPhOPA	Hydroxy-3-phenoxypropyl acrylate
74	iOA	Isooctyl acrylate
75	PDA	1,4-Phenylene diacrylate
76	PETA	Pentaerythritol tetraacrylate
77	TEGDA	Tetra(ethylene glycol) diacrylate
78	GDMA	Glycerol dimethacrylate
79	TCDMDA	Tricyclodecane-dimethanol diacrylate
80	MAHBP	4-Methacryloxy-2-hydroxybenzophenone
81	BTHPhMA	Benzotriazol-2-yl)-4-hydroxyphenyl]ethyl methacrylate
82	NGPDA	Neopentyl glycol propoxylate diacrylate
83	DMEMAm	N-[2-(N,N-Dimethylamino)ethyl]methacrylamide
84	DEAEA	Diethylamino ethyl acrylate
85	pEGPhEA	Poly(ethylene glycol) phenyl ether acrylate
86	PhEMA	2-Phenylethyl methacrylate
87	pPGDMA	Poly(propylene glycol) (400) dimethacrylate
88	MAAHS	Methacrylic acid N-hydroxysuccinimide ester
89	HPHPBAH	Hydroxypivalyl hydroxypivalate bis[6-(acryloyloxy)hexanoate]
90	PFPhA	Pentafluorophenyl acrylate
91	DEAEMA	Diethylaminoethyl methacrylate
92	TBPhA	2,4,6-Tribromophenyl acrylate
93	PMMA	1-Pyrenylmethyl methacrylate
94	MAPU	2-methacryloxyethyl phenyl urethane
95	NBMA	Norbornyl methacrylate
96	PhMAm	N-Phenylmethacrylamide
97	DEGEHA	Di(ethylene glycol) 2-ethylhexyl ether acrylate
98	HBMA	Hydroxybutyl methacrylate
99	pPGMEA	Poly(propylene glycol) methyl ether acrylate
100	iDMA	Isodecyl methacrylate
101	DiPEMA	2-Diisopropylaminoethyl methacrylate
102	AEMAm.C	N-(2-aminoethyl) methacrylamide hydrochloride

103	HPMAP	Hydroxypropyl 2-(methacryloyloxy)ethyl phthalate
104	MTEMA	Methylthioethyl methacrylate
105	PEDAM	Pentaerythritol diacrylate monostearate
106	MHMB	Methyl 3-hydroxy-2-methylenebutyrate
107	EG3DMA	Tri(ethylene glycol) dimethacrylate
108	HDFHUA	Heptadecafluoro-2-hydroxyundecyl acrylate
109	HPA	Hydroxypropyl acrylate
110	NaPhA	Sodium 3-phenyl-acrylate
111	ZrCEA	Zirconium carboxyethyl acrylate
112	BPEODA	Bisphenol A ethoxylate diacrylate
113	COEA	2-Cinnamoyloxyethyl acrylate
114	DEGDMA	Diethylene glycol dimethacrylate
115	OFHMA	Octafluoro-2-hydroxy-6-(trifluoromethyl)heptyl methacrylate
116	iBuMA	Isobutyl methacrylate
117	GMA	Glycidyl methacrylate
118	iDA	Isodecyl acrylate
119	SPAK	Sulfopropyl acrylate potassium salt
120	BFEODA	Bisphenol F ethoxylate diacrylate
121	BnPA	Benzyl 2-n-propyl acrylate
122	CzEA	Carbazol-9-yl ethyl acrylate
123	tBCHMA	Tertbutylcyclohexyl methacrylate
124	TFPMA	Tetrafluoropropyl methacrylate
125	MA	Methyl acrylate
126	TDFOcA	Tridecafluorooctyl acrylate
127	MAETA	4-Methacryloxyethyl trimellitic anhydride
128	DVSeb	Divinyl sebacate
129	TMPOTA	Trimethylolpropane propoxylate triacrylate
130	BMENBC	Bis(2-methacryloxyethyl) N,N’-1,9-nonylene biscarbamate
131	NBnMA	o-Nitrobenzyl methacrylate
132	nOcMA	n-Octyl methacrylate,
133	HFHUMA	Hexadecafluoro-2-hydroxy-10-(trifluoromethyl)undecyl methacrylate
134	MAEP	Monoacryloxyethyl phosphate
135	CHA	Cyclohexyl acrylate
136	iBOA	Isobornyl acrylate
137	THFuMA	Tetrahydrofurfuryl methacrylate
138	DMAEA	Dimethylamino-ethyl acrylate
139	PhEA	2-Phenylethyl acrylate
140	PAHEMA	Phosphoric acid 2-hydroxyethyl methacrylate ester
141	BOEMA	Butoxyethyl methacrylate
142	HDFDA	Heptadecafluorodecyl acrylate
143	HFiPMA	Hexafluoroisopropyl methacrylate
144	BMA	Butyl methacrylate
145	DMPAm	N-[3-(Dimethylamino)propyl]acrylamide
146	GDGDA	Glycerol 1,3-diglycerolate diacrylate
147	EHMA	Ethylhexyl methacrylate
148	DFFMOA	Dodecafluoro-7-(trifluoromethyl)-octyl acrylate
149	BAC	N,N'-Bis(acryloyl)cystamine
150	HEAm	N-Hydroxyethyl acrylamide
151	mMAOES	mono-2-(Methacryloyloxy)ethyl succinate
152	BA	Butyl acrylate
153	BMAM	N-Benzylmethacrylamide
154	FMHPNMA	Trifluoro-2′-(trifluoromethyl)-2′-hydroxy)propyl]-3-norbornyl methacrylate

155	MMA	Methyl methacrylate
156	BACOEA	Butylamino carbonyl oxy ethyl acrylate
157	EOEA	Ethoxyethyl acrylate
158	iBA	Isobutyl acrylate
159	SPMAK	3-Sulfopropyl methacrylate potassium salt
160	DHPA	2,3-dihydroxypropyl acrylate
161	F6BMA	Hexafluorobutyl methacrylate
	IBESMA	1,7,7-trimethylbicyclo[2.2.1]heptan-2-yl 6-(methacryloyloxy)-4-oxohexanoate
162	HDFDMA	Heptadecafluorodecyl methacrylate
163	TFCAm	7-[4-(Trifluoromethyl)coumarin]acrylamide
164	AODMBA	(R)-α-Acryloyloxy-β,β-dimethyl-γ-butyrolactone
165	PA	Propargyl acrylate
166	OFPMA	Octafluoropentyl methacrylate
167	iBOMAm	N-(Isobutoxymethyl)acrylamide
168	BAPA	1,4-Bis(acryloyl)piperazine
169	DFHNMA	Dodecafluoro-2-hydroxy-8-(trifluoromethyl)nonyl methacrylate
170	F6BA	Hexafluorobutyl acrylate
171	MAEPC	2-Methacryloyloxyethyl phosphorylcholine
172	pPGNEA	Poly(propylene glycol) 4-nonylphenyl ether acrylate
173	SEMA	2-Sulfoethyl methacrylate
174	VMA	Vinyl methacrylate
175	HMA	Hexyl methacrylate
176	EbCNA	Ethyl-cis-B-cyano-acrylate
177	THMMAm	N-[Tris(hydroxymethyl)methyl]acrylamide
178	HA	Hexyl acrylate
179	tBMAm	N-tert-Butylmethacrylamide
180	HTFDA	Hexadecafluoro-9-(trifluoromethyl)decyl acrylate
181	AMA	Allyl methacrylate
182	EEMA	Ethoxyethyl methacrylate
183	EHA	Ethylhexyl acrylate
184	PMAm	N-(Phthalimidomethyl)acrylamide
185	tBMA	Tert-butyl methacrylate
186	TMBAm	N-(1,1,3,3-Tetramethylbutyl)acrylamide
187	DEGMA	Di(ethylene glycol) methyl ether methacrylate
188	TBPMA	Tribromophenyl methacrylate
189	EGMMA	Ethylene glycol methyl ether methacrylate
190	EEA	Ethyl 2-ethylacrylate
191	LMA	Lauryl methacrylate
192	MPDSAH	Methacryloylamino)propyl]dimethyl(3-sulfopropyl)ammonium hydroxide inner salt
193	AnMA	Anthracenylmethylacrylate
194	EBAM	N,N'-Ethylenebisacrylamide
195	F7BA	Heptafluorobutyl acrylate
196	HFDA	Heneicosafluorododecyl acrylate
197	HPMAm	N-(2-Hydroxypropyl)methacrylamide
198	MEDMSAH	[2-(Methacryloyloxy)ethyl]dimethyl-(3-sulfopropyl) ammonium hydroxide
199	PBPhMA	Pentabromophenyl methacrylate
200	PFPhMA	Pentafluorophenyl methacrylate
201	HBA	Hydroxybutyl acrylate
202	PPPDMA	PEO(5800)-b-PPO(3000)-b-PEO(5800) dimethacrylate
203	tBAm	N-tert-Butylacrylamide
204	CEA	Carboxyethyl acrylate
205	HfCEA	Hafnium carboxyethyl acrylate

206	TMPDAE	Trimethyl propane diallyl ether
207	TPhMAm	N-(Triphenylmethyl)methacrylamide
208	DAAM	N,N-Diallylacrylamide
209	EMA	Ethyl methacrylate
210	EPA	Ethyl 2-propylacrylate
211	HMBMAm	N,N'-Hexamethylenebis(methacrylamide)
212	TDFOMA	Tridecafluorooctyl methacrylate
213	DRA	Disperse red 1 acrylate
214	HPhMA	N-(4-Hydroxyphenyl)methacrylamide
215	MAL	Methacryloyl-L-Lysine
216	NDMAm	N-Dodecylmethacrylamide
217	PBBA	Pentabromobenzyl acrylate
218	pEGMEMA	Poly(ethylene glycol) methyl ether methacrylate
219	DMMAm	N,N-Dimethylmethacrylamide
220	DYA	Disperse yellow 7 acrylate
221	EG4DMA	Tetraethylene glycol dimethacrylate
222	Mam	Methacrylamide
223	pPGA	Poly(propylene glycol) acrylate
224	DOAm	Disperse Orange 3 acrylamide
225	HPMA	Hydroxypropyl methacrylate
226	BOMAm	N-(Butoxymethyl)acrylamide
227	NMEMA	2-N-Morpholinoethyl methacrylate
228	tBA	Tert-butyl acrylate
229	BMAOEP	Bis[2-(methacryloyloxy)ethyl] phosphate
230	ECNTA	Ethyl-2-cyano-3-(2-thienyl)acrylate
231	F7BMA	Heptafluorobutyl methacrylate
232	NAM	N-Acryloylmorpholine
233	pEGMEA	Poly(ethylene glycol) methyl ether acrylate
234	PFPMA	Pentafluoropropyl methacrylate
235	EGMEA	Ethylene glycol methyl ether acrylate
236	HMAm	N-(Hydroxymethyl)acrylamide
237	iPAM	N-Isopropylacrylamide
238	GMMA	Glycerol monomethacrylate
239	AAm	Acrylamide
240	MMAm	N-Methylmethacrylamide
241	PMA	Propargyl methacrylate
242	ZrA	Zirconium acrylate
243	AA	Allyl acrylate
244	CMAOE	Caprolactone 2-(methacryloyloxy)ethyl ester
245	DMAm	N,N'-Dimethylacrylamide
246	EGPhMA	Ethylene glycol phenyl ether methacrylate
247	HEA	Hydroxyethyl acrylate
248	MBMAm	N,N'-Methylenebismethacrylamide
249	NAS	N-Acryloxysuccinimide
250	tBOCAPAm	N-(t-BOC-aminopropyl)methacrylamide
251	TEGMA	Tri(ethylene glycol) methyl ether methacrylate
252	ZnA	Zinc acrylate
253	HMBAM	N,N’-Hexamethylenebisacrylamide
254	PBPhA	Pentabromophenyl acrylate
255	PFPA	Pentafluoropropyl acrylate
256	AAcAm	Diacetone acrylamide
257	AcAPAm	N-[2-(Acryloylamino)phenyl]acrylamide
258	DHEBAM	N,N'-(1,2-Dihydroxyethylene)bisacrylamide

259	EA	Ethyl acrylate
260	EaNIA	Ethyl trans-a-cyano-3-indole-acrylate
261	GA	Glycidyl acrylate
262	MAA	Methyl 2-acetamidoacrylate
263	MBAm	N,N'-Methylenebisacrylamide
264	MOPAm	N-(3-Methoxypropyl)acrylamide
265	NPhPMA	Nitrophenyl-2-pyrrolidonemethyl acrylate
266	pEGDA	Polyethylene glycol diacrylate
267	pEGMA	Poly(ethylene glycol) methacrylate
268	PPDDA	3-phenoxypropane-1,2-diyl diacrylate
269	SMA	Stearyl methacrylate
270	tBEMAm	N-(3,3-dimethylbutyl)methacrylamide
271	pFDA	Perfluorodecyl acrylate
272	CHPMA	Chloro-2-hydroxy-propyl methacrylate
273	BPDMA	Bisphenol A dimethacrylate

Table S2: hPSC attachment on monomer screen at 24 h ranked (high to low) by total cell number (DAPI nuclei count) and OCT4+ nuclei count.

	DAPI count			OCT4+ count		
Rank order	Polymer ID	Mean	STDEV	Polymer ID	Mean	STDEV
1	**13BDDA**	60	44	**13BDDA**	56	41
2	**THFuA**	55	35	**THFuA**	52	32
3	**EGDPEA**	51	22	**EGDPEA**	48	21
4	**MAAH**	49	20	**MAAH**	47	20
5	**MAPtMA**	43	39	**MAPtMA**	40	36
6	**TMPETA**	42	26	**MAEA**	38	25
7	**TMOBDA**	40	22	**13BDDMA**	38	53
8	**BDDA**	40	43	**EGDA**	37	68
9	**MAEA**	39	26	**TMPETA**	37	23
10	**13BDDMA**	39	55	**TMCHMA**	36	32
11	**TMCHMA**	38	34	**TMOBDA**	36	20
12	**EGDA**	38	70	**DMAEMA**	36	37
13	**DMAEMA**	37	37	**BDDA**	34	38
14	**DVAd**	35	18	**SolA**	32	31
15	**E3GDA**	35	26	**HBOPBA**	31	15
16	**SolA**	35	34	**E3GDA**	31	25
17	**PDDMA**	34	26	**DVAd**	31	16
18	**DMEMAm**	33	24	**PDDMA**	30	24
19	**HBOPBA**	32	14	**TAHTA**	30	25
20	**TAHTA**	31	26	**CNEA**	29	22
21	**MAEACl**	31	21	**EGDCMA**	28	22
22	**DMAPA**	30	32	**OFPA**	28	25
23	**CNEA**	30	24	**DPEPHA**	27	31
24	**EGDCMA**	29	22	**ZrBNCTA**	26	16
25	**OFPA**	29	26	**HEODA**	26	28

26	**DPEPHA**	29	33	**HDMPDA**	26	11
27	**HDMPDA**	28	12	**PETrA**	25	26
28	**HEODA**	28	30	**MAEACl**	25	22
29	**PETrA**	28	30	**DEGDA**	24	15
30	**ZrBNCTA**	27	17	**NpMA**	24	13
31	**DEGDA**	27	17	**TBNpMA**	24	14
32	**NpMA**	26	15	**14BDDMA**	24	24
33	**tBAEMA**	26	51	**TMHA**	24	43
34	**14BDDMA**	26	26	**mMAOEM**	23	15
35	**TBNpMA**	25	15	**DMAPA**	23	30
36	**NGDA**	24	25	**DDDMA**	23	21
37	**TMHA**	24	45	**NGDA**	23	24
38	**mMAOEM**	24	16	**TAIC**	22	29
39	**DMPMAm**	23	9	**tBAEMA**	22	43
40	**NpA**	23	22	**NpA**	21	20
41	**DDDMA**	23	21	**EGPEA**	21	20
42	**TAIC**	22	29	**AEMA.C**	20	22
43	**EGPEA**	22	20	**LaA**	19	22
44	**TMPTA**	22	21	**BAPODA**	19	16
45	**AEMA.C**	22	24	**APMAm.C**	19	30
46	**HFiPA**	21	20	**HFPDA**	19	12
47	**DFHA**	20	12	**tBCHA**	18	20
48	**APMAm.C**	20	32	**TMPTA**	18	18
49	**NDDMA**	20	19	**DFHA**	18	11
50	**tBCHA**	20	21	**AOHPMA**	17	21
51	**BAPODA**	20	16	**EGDMA**	17	20
52	**LaA**	19	22	**NDDMA**	17	16
53	**HFPDA**	19	12	**PhA**	17	24
54	**BzHPEA**	19	19	**TPGDA**	17	21
55	**TPGDA**	19	21	**BnA**	17	15
56	**BnA**	19	17	**HDDMA**	17	13
57	**PhA**	18	25	**FuMA**	17	27
58	**HDDMA**	18	15	**BzHPEA**	17	16
59	**pEGDMA**	18	17	**ExA**	16	26
60	**EGDMA**	18	21	**CHMA**	16	16
61	**FuMA**	18	29	**TMOPTMA**	16	11
62	**PhMA**	18	21	**BPAPGDA**	16	26
63	**AOHPMA**	18	21	**iBMA/tBAEMA**	16	27
64	**CHMA**	17	16	**PhMA**	15	19
65	**BAGDA**	17	25	**BHMA**	15	19
66	**ExA**	16	26	**pEGDMA**	15	15
67	**BHMOPhP**	16	12	**DEGEEA**	15	22
68	**TMOPTMA**	16	12	**BAGDA**	15	22
69	**iBMA/tBAEMA**	16	27	**ODA**	15	8
70	**ODA**	16	9	**iCEMA**	14	12
71	**BHMA**	16	19	**BHMOPhP**	14	11

72	**BPAPGDA**	16	27	**DMPMAm**	14	7
73	**GPOTA**	16	9	**GPOTA**	14	8
74	**iBMA**	15	29	**HFiPA**	14	16
75	**BnMA**	15	14	**iBMA**	13	25
76	**DEGEEA**	15	22	**BnMA**	13	12
77	**iCEMA**	15	12	**HPhOPA**	13	18
78	**NGPDA**	15	18	**iOA**	13	11
79	**DMA**	15	19	**PDA**	13	9
80	**MAHBP**	15	7	**PETA**	13	7
81	**HDMA**	14	26	**TEGDA**	13	18
82	**PETA**	14	8	**GDMA**	13	9
83	**DEAEA**	14	20	**HDMA**	13	24
84	**HPhOPA**	14	20	**TCDMDA**	13	17
85	**PHPMA**	14	17	**MAHBP**	12	7
86	**TCDMDA**	14	19	**BTHPhMA**	12	11
87	**PDA**	14	10	**DMA**	12	17
88	**GDMA**	13	10	**NGPDA**	12	14
89	**iOA**	13	12	**DMEMAm**	12	11
90	**TEGDA**	13	19	**DEAEA**	12	17
91	**MAAHS**	13	23	**pEGPhEA**	12	17
92	**HPHPBAH**	13	10	**PhEMA**	12	19
93	**BTHPhMA**	13	11	**pPGDMA**	12	13
94	**TBPhA**	13	13	**MAAHS**	12	20
95	**HEODA/EEMA**	12	22	**PPDDA**	11	14
96	**PhEMA**	12	20	**PHPMA**	11	14
97	**pEGPhEA**	12	18	**HEODA/EEMA**	11	20
98	**PPDDA**	12	15	**HPHPBAH**	11	9
99	**pPGDMA**	12	13	**PFPhA**	11	9
100	**iDMA**	12	20	**DEAEMA**	11	12
101	**HBMA**	11	34	**TBPhA**	11	13
102	**PFPhA**	11	9	**PMMA**	11	15
103	**DEGEHA**	11	16	**MAPU**	10	12
104	**MAPU**	11	14	**NBMA**	10	15
105	**NBMA**	11	15	**PhMAm**	10	14
106	**PhMAm**	11	15	**DEGEHA**	10	15
107	**PMMA**	11	15	**HBMA**	10	30
108	**DEAEMA**	11	13	**pPGMEA**	10	14
109	**pPGMEA**	11	15	**iDMA**	10	17
110	**HPA**	10	17	**DiPEMA**	10	11
111	**PEDAM**	10	24	**AEMAm.C**	9	15
112	**MTEMA**	10	13	**HPMAP**	9	12
113	**DiPEMA**	10	11	**BPDMA**	9	10
114	**AEMAm.C**	10	16	**MTEMA**	9	11
115	**HPMAP**	10	12	**PEDAM**	8	19
116	**EG3DMA**	9	15	**MHMB**	8	8
117	**BPDMA**	9	10	**EG3DMA**	8	13

118	**HDFHUA**	9	15	**HDFHUA**	8	14
119	**COEA**	9	12	**HPA**	8	16
120	**DEGDMA**	9	9	**NaPhA**	8	8
121	**DMPAm**	9	12	**ZrCEA**	8	8
122	**iDA**	9	11	**BPEODA**	8	9
123	**MHMB**	9	8	**COEA**	8	11
124	**BPEODA**	9	10	**DEGDMA**	8	8
125	**NaPhA**	8	8	**OFHMA**	8	8
126	**iBuMA**	8	10	**iBuMA**	7	9
127	**SPAK**	8	6	**GMA**	7	14
128	**ZrCEA**	8	8	**iDA**	7	9
129	**OFHMA**	8	8	**SPAK**	7	6
130	**TDFOcA**	8	8	**BFEODA**	7	7
131	**GMA**	7	14	**BnPA**	7	7
132	**MA**	7	10	**CzEA**	7	7
133	**TFPMA**	7	10	**tBCHMA**	7	6
134	**BFEODA**	7	7	**TFPMA**	7	10
135	**BnPA**	7	7	**MA**	7	9
136	**MAETA**	7	9	**TDFOcA**	7	7
137	**tBCHMA**	7	7	**MAETA**	6	9
138	**CzEA**	7	7	**DVSeb**	6	6
139	**SMA**	7	10	**TMPOTA**	6	12
140	**BMENBC**	7	2	**BMENBC**	6	2
141	**DVSeb**	7	6	**NBnMA**	6	18
142	**HFHUMA**	7	5	**nOcMA**	6	9
143	**PAHEMA**	7	7	**SMA**	6	9
144	**HDFDA**	6	8	**HFHUMA**	6	5
145	**TMPOTA**	6	12	**MAEP**	6	8
146	**nOcMA**	6	9	**CHA**	5	8
147	**iBOA**	6	7	**iBOA**	5	6
148	**NBnMA**	6	18	**THFuMA**	5	7
149	**PhEA**	6	14	**DMAEA**	5	11
150	**BOEMA**	6	8	**PhEA**	5	12
151	**BMA**	6	10	**PAHEMA**	5	5
152	**THFuMA**	6	7	**BOEMA**	5	7
153	**CHA**	6	9	**HDFDA**	5	5
154	**MAEP**	6	8	**HFiPMA**	5	7
155	**GDGDA**	5	5	**BMA**	5	8
156	**HEAm**	5	7	**DMPAm**	5	6
157	**DMAEA**	5	11	**GDGDA**	5	4
158	**BMAM**	5	6	**EHMA**	4	4
159	**HFiPMA**	5	7	**DFFMOA**	4	7
160	**DFFMOA**	5	8	**BAC**	4	5
161	**BACOEA**	5	6	**HEAm**	4	6
162	**EHMA**	4	4	**mMAOES**	4	4
163	**BAC**	4	5	**BA**	4	8

164	**mMAOES**	4	4	**BMAM**	4	6
165	**pPGDA**	4	5	**FMHPNMA**	4	4
166	**BA**	4	8	**MMA**	4	6
167	**MMA**	4	6	**CHPMA**	4	7
168	**FMHPNMA**	4	4	**pPGDA**	4	4
169	**SPMAK**	4	7	**BACOEA**	4	5
170	**CHPMA**	4	7	**EOEA**	4	6
171	**DHPA**	4	6	**iBA**	4	3
172	**PMAm**	4	6	**SPMAK**	3	6
173	**OFPMA**	4	7	**DHPA**	3	6
174	**EOEA**	4	6	**F6BMA**	3	3
175	**iBA**	4	3	**pFDA**	3	3
176	**pFDA**	4	3	**IBESMA**	3	4
177	**TFCAm**	4	5	**HDFDMA**	3	4
178	**F6BMA**	3	3	**TFCAm**	3	5
179	**IBESMA**	3	4	**AODMBA**	3	4
180	**HDFDMA**	3	4	**PA**	3	6
181	**PA**	3	7	**OFPMA**	3	5
182	**EbCNA**	3	7	**iBOMAm**	3	2
183	**AODMBA**	3	4	**BAPA**	3	3
184	**pPGNEA**	3	6	**DFHNMA**	3	3
185	**iBOMAm**	3	2	**F6BA**	3	4
186	**HMA**	3	6	**MAEPC**	3	5
187	**SEMA**	3	5	**pPGNEA**	3	6
188	**BAPA**	3	3	**SEMA**	3	5
189	**DFHNMA**	3	3	**VMA**	3	2
190	**F6BA**	3	4	**HMA**	2	5
191	**MAEPC**	3	5	**EbCNA**	2	6
192	**EEMA**	3	5	**THMMAm**	2	4
193	**tBMAm**	3	3	**HA**	2	3
194	**VMA**	3	2	**tBMAm**	2	3
195	**HA**	2	3	**HTFDA**	2	3
196	**EHA**	2	6	**AMA**	2	4
197	**THMMAm**	2	4	**EEMA**	2	5
198	**AMA**	2	4	**EHA**	2	5
199	**EGMMA**	2	4	**FuMA/tBAEMA**	2	2
200	**HTFDA**	2	3	**PMAm**	2	3
201	**DEGMA**	2	3	**tBMA**	2	4
202	**FuMA/tBAEMA**	2	2	**TMBAm**	2	4
203	**tBMA**	2	4	**DEGMA**	2	3
204	**TMBAm**	2	4	**TBPMA**	2	3
205	**TBPMA**	2	3	**EGMMA**	2	3
206	**HFDA**	2	3	**EEA**	2	2
207	**LMA**	2	2	**LMA**	2	2
208	**tBAm**	2	4	**MPDSAH**	2	2
209	**AnMA**	2	2	**AnMA**	2	2

210	**EEA**	2	2	**EBAM**	1	2
211	**MPDSAH**	2	2	**F7BA**	1	2
212	**EBAM**	2	2	**HFDA**	1	3
213	**PFPhMA**	2	3	**HPMAm**	1	3
214	**F7BA**	1	2	**MEDMSAH**	1	2
215	**HPMAm**	1	3	**PBPhMA**	1	2
216	**MEDMSAH**	1	2	**PFPhMA**	1	3
217	**PBPhMA**	1	2	**HBA**	1	3
218	**TMPDAE**	1	4	**PPPDMA**	1	2
219	**DAAM**	1	2	**tBAm**	1	3
220	**HBA**	1	3	**CEA**	1	3
221	**MAL**	1	2	**HfCEA**	1	3
222	**NDMAm**	1	2	**TMPDAE**	1	4
223	**PPPDMA**	1	2	**TPhMAm**	1	2
224	**CEA**	1	3	**DAAM**	1	1
225	**HfCEA**	1	3	**EMA**	1	3
226	**PBBA**	1	4	**EPA**	1	2
227	**TPhMAm**	1	2	**HMBMAm**	1	3
228	**EMA**	1	3	**TDFOMA**	1	2
229	**EPA**	1	2	**DRA**	1	2
230	**DYA**	1	3	**HPhMA**	1	2
231	**HMBMAm**	1	3	**MAL**	1	2
232	**pEGMEMA**	1	2	**NDMAm**	1	1
233	**TDFOMA**	1	2	**PBBA**	1	3
234	**DRA**	1	2	**pEGMEMA**	1	2
235	**HPhMA**	1	2	**DMMAm**	1	2
236	**pPGA**	1	1	**DYA**	1	2
237	**DHEBAM**	1	3	**EG4DMA**	1	1
238	**DMMAm**	1	2	**Mam**	1	2
239	**EG4DMA**	1	1	**pPGA**	1	1
240	**Mam**	1	2	**DOAm**	1	1
241	**DOAm**	1	1	**HPMA**	1	1
242	**HPMA**	1	1	**BOMAm**	1	2
243	**BOMAm**	1	2	**NMEMA**	1	1
244	**NMEMA**	1	1	**tBA**	1	1
245	**tBA**	1	1	**BMAOEP**	1	2
246	**BMAOEP**	1	2	**ECNTA**	1	1
247	**ECNTA**	1	1	**F7BMA**	1	2
248	**EGMEA**	1	1	**NAM**	1	2
249	**F7BMA**	1	2	**pEGMEA**	1	1
250	**NAM**	1	2	**PFPMA**	1	1
251	**pEGMEA**	1	1	**EGMEA**	1	1
252	**PFPMA**	1	1	**HMAm**	1	2
253	**ZnA**	1	1	**iPAM**	1	1
254	**HMAm**	1	2	**GMMA**	1	1
255	**iPAM**	1	1	**AAm**	0	1

256	**ZrA**	1	1	**MMAm**	0	1
257	**GMMA**	1	1	**PMA**	0	1
258	**AAm**	0	1	**tBEMAm**	0	1
259	**MMAm**	0	1	**ZrA**	0	1
260	**PMA**	0	1	**AA**	0	1
261	**tBEMAm**	0	1	**CMAOE**	0	1
262	**AA**	0	1	**DMAm**	0	1
263	**CMAOE**	0	1	**EGPhMA**	0	1
264	**DMAm**	0	1	**HEA**	0	1
265	**EGPhMA**	0	1	**MBMAm**	0	1
266	**HEA**	0	1	**NAS**	0	1
267	**MBMAm**	0	1	**tBOCAPAm**	0	1
268	**NAS**	0	1	**TEGMA**	0	1
269	**tBOCAPAm**	0	1	**ZnA**	0	1
270	**TEGMA**	0	1	**HMBAM**	0	1
271	**HMBAM**	0	1	**PBPhA**	0	1
272	**PBPhA**	0	1	**PFPA**	0	1
273	**PFPA**	0	1	**AAcAm**	0	0
274	**pEGDA**	0	0	**AcAPAm**	0	0
275	**AAcAm**	0	0	**DHEBAM**	0	0
276	**AcAPAm**	0	0	**EA**	0	0
277	**EA**	0	0	**EaNIA**	0	0
278	**EaNIA**	0	0	**GA**	0	0
279	**GA**	0	0	**MAA**	0	0
280	**MAA**	0	0	**MBAm**	0	0
281	**MBAm**	0	0	**MOPAm**	0	0
282	**MOPAm**	0	0	**NPhPMA**	0	0
283	**NPhPMA**	0	0	**pEGDA**	0	0
284	**pEGMA**	0	0	**pEGMA**	0	0

Table S3 hPSC attachment on co-polymer arrays after 24 h ranked (high-low) by OCT4+ nuclei count

Rank order*	Letter ID	Polymer ID	Average OCT4 count	STDEV
1	**D:O**	**TCDMDA:MAETA**	164	80
2	**P:E**	**THFuA:EG4DMA**	153	153
3	**F:G**	**BDDA:EGDA**	151	90
4	**H:Q**	**GDMA:BA**	135	119
5	**P:B**	**THFuA:NGDA**	134	127
6	**P:C**	**THFuA:BHMOPhP**	131	122
7	**P:H**	**THFuA:GDMA**	129	108
8	**O:D**	**MAETA:TCDMDA**	124	96
9	**G:F**	**EGDA:BDDA**	123	85
10	**H:F**	**GDMA:BDDA**	123	87
11	**B:J**	**NGDA:mMAOES**	122	70
12	**D:G**	**TCDMDA:EGDA**	120	74
13	**F:P**	**BDDA:THFuA**	117	96
14	**H:D**	**GDMA:TCDMDA**	117	94
15	**M:C**	**EGDPEA:BHMOPhP**	115	87
16	**P**	**THFuA**	112	154
17	**C**	**BHMOPhP**	112	141
18	**D:N**	**TCDMDA:FuMA**	112	103
19	**I:B**	**DEAMEA:NGDA**	111	90
20	**L:D**	**HEMA:TCDMDA**	110	91
21	**D:P**	**TCDMDA:THFuA**	110	57
22	**L:E**	**HEMA:EG4DMA**	109	145
23	**E:A**	**EG4DMA:HBOPBA**	104	71
24	**D:Q**	**TCDMDA:BA**	104	102
25	**H:R**	**GDMA:TDFOMA**	103	106
26	**D**	**TCDMDA**	102	85
27	**D:B**	**TCDMDA:NGDA**	102	125
28	**F:J**	**BDDA:mMAOES**	101	54
29	**M:E**	**EGDPEA:EG4DMA**	101	101
30	**P:N**	**THFuA:FuMA**	100	89
31	**D:J**	**TCDMDA:mMAOES**	98	46
32	**H:C**	**GDMA:BHMOPhP**	98	64
33	**H:N**	**GDMA:FuMA**	98	41
34	**F:O**	**BDDA:MAETA**	98	51
35	**O:B**	**MAETA:NGDA**	98	127
36	**G**	**EGDA**	97	86
37	**F:K**	**BDDA:tBAEMA**	95	141
38	**L:B**	**HEMA:NGDA**	95	145
39	**B:F**	**NGDA:BDDA**	94	83
40	**H:B**	**GDMA:NGDA**	94	74
41	**B:H**	**NGDA:GDMA**	94	68
42	**B:L**	**NGDA:HEMA**	94	52

43	**O:A**	**MAETA:HBOPBA**	93	132
44	**N:F**	**FuMA:BDDA**	93	93
45	**R:G**	**TDFOMA:EGDA**	92	192
46	**P:F**	**THFuA:BDDA**	90	88
47	**H:G**	**GDMA:EGDA**	90	50
48	**O:H**	**MAETA:GDMA**	90	83
49	**I:E**	**DEAMEA:EG4DMA**	90	79
50	**N**	**FuMA**	90	96
51	**F:E**	**BDDA:EG4DMA**	88	66
52	**Q:E**	**BA:EG4DMA**	87	132
53	**D:C**	**TCDMDA:BHMOPhP**	87	74
54	**N:D**	**FuMA:TCDMDA**	86	85
55	**A:B**	**HBOPBA:NGDA**	86	107
56	**P:D**	**THFuA:TCDMDA**	85	65
57	**H:K**	**GDMA:tBAEMA**	85	57
58	**M:B**	**EGDPEA:NGDA**	84	73
59	**G:C**	**EGDA:BHMOPhP**	83	109
60	**N:I**	**FuMA:DEAEMA**	82	82
61	**P:R**	**THFuA:TDFOMA**	82	67
62	**N:C**	**FuMA:BHMOPhP**	82	72
63	**F:H**	**BDDA:GkDMA**	81	68
64	**G:D**	**EGDA:TCDMDA**	80	80
65	**R:B**	**TDFOMA:NGDA**	80	173
66	**D:F**	**TCDMDA:BDDA**	79	45
67	**B:D**	**NGDA:TCDMDA**	78	97
68	**H:P**	**GDMA:THFuA**	78	72
69	**M:Q**	**EGDPEA:BA**	78	98
70	**M:I**	**EGDPEA:DEAEMA**	77	89
71	**A:G**	**HBOPBA:EGDA**	76	74
72	**C:D**	**BHMOPhP:TCDMDA**	76	83
73	**H:A**	**GDMA:HBOPBA**	73	64
74	**B:O**	**NGDA:MAETA**	72	45
75	**N:H**	**FuMA:GDMA**	72	48
76	**N:E**	**FuMA:EG4DMA**	72	47
77	**F:M**	**BDDA:EGDPEA**	70	82
78	**F**	**BDDA**	70	68
79	**M:R**	**EGDPEA:h TDFOMA**	69	116
80	**O:F**	**MAETA:BDDA**	68	57
81	**B:I**	**NGDA:DEAEMA**	68	40
82	**P:Q**	**THFuA:BA**	68	107
83	**I:D**	**DEAMEA:TCDMDA**	68	38
84	**G:O**	**EGDA:MAETA**	67	75
85	**B:M**	**NGDA:EGDPEA**	67	75
86	**H:E**	**GDMA:EG4DlMA**	66	54
87	**L:M**	**HEMA:EGDPEA**	65	68

88	**A:C**	**HBOPBA:BHMOPhP**	65	63
89	**F:D**	**BDDA:TCDMDA**	65	64
90	**F:I**	**BDDA:DEAEMA**	65	45
91	**H:O**	**GDMA:MAETA**	64	80
92	**O:E**	**MAETA:EG4DMA**	63	55
93	**M:F**	**EGDPEA:BDDA**	62	69
94	**J:B**	**mMAOES:NGDA**	62	110
95	**D:L**	**TCDMDA:HEMA**	62	51
96	**B**	**NGDA**	62	75
97	**F:B**	**BDDA:NGDA**	62	51
98	**H:I**	**GDMA:DEAEMA**	61	76
99	**C:B**	**BHMOPhP:NGDA**	61	74
100	**D:M**	**TCDMDA:EGDPEA**	61	74
101	**F:L**	**BDDA:HEMA**	60	58
102	**D:A**	**TCDMDA:HBOPBA**	60	48
103	**E:F**	**EG4DMA:BDDA**	59	63
104	**G:A**	**EGDA:HBOPBA**	59	129
105	**B:G**	**NGDA:EGDA**	59	71
106	**N:Q**	**FuMA:BA**	59	127
107	**H:M**	**GDMA:EGDPEA**	59	45
108	**O:G**	**MAETA:EGDA**	59	64
109	**E:B**	**EG4DMA:NGDA**	58	64
110	**M:H**	**EGDPEA:GDMA**	57	76
111	**E:G**	**EG4DMA:EGDA**	57	56
112	**A**	**HBOPBA**	56	43
113	**A:F**	**HBOPBA:BDDA**	56	58
114	**D:H**	**TCDMDA:GDMA**	56	52
115	**N:K**	**FuMA:tBAEMA**	56	38
116	**F:C**	**BDDA:BHMOPhP**	56	56
117	**A:D**	**HBOPBA:TCDMDA**	56	53
118	**F:R**	**BDDA:TDFOMA**	55	46
119	**G:J**	**EGDA:mMAOES**	55	67
120	**D:I**	**TCDMDA:DEAEMA**	55	49
121	**P:G**	**THFuA:EGDA**	55	73
122	**L:A**	**HEMA:HBOPBA**	55	56
123	**A:E**	**HBOPBA:EG4DMA**	55	70
124	**M:D**	**EGDPEA:TCDMDA**	54	53
125	**M:K**	**EGDPEA:tBAEMA**	54	71
126	**Q:D**	**BA:TCDMDA**	54	63
127	**I:F**	**DEAMEA:BDDA**	54	70
128	**J:D**	**mMAOES:TCDMDA**	53	37
129	**K:O**	**tBAEMA:MAETA**	53	41
130	**R:F**	**TDFOMA:BDDA**	53	65
131	**M:A**	**EGDPEA:HBOPBA**	53	45
132	**A:H**	**HBOPBA:GDMA**	52	84
133	**Q:B**	**BA:NGDA**	52	52

134	**B:C**	**NGDA:BHMOPhP**	52	25
135	**E:C**	**EG4DMA:BHMOPhP**	52	51
136	**I:H**	**DEAMEA:GDMA**	51	122
137	**O:M**	**MAETA:EGDPEA**	51	62
138	**C:J**	**BHMOPhP:mMAOES**	49	52
139	**C:L**	**BHMOPhP:HEMA**	48	84
140	**C:F**	**BHMOPhP:BDDA**	48	61
141	**M:O**	**EGDPEA:MAETA**	48	89
142	**C:E**	**BHMOPhP:EG4DMA**	47	49
143	**D:E**	**TCDMDA:EG4DMA**	47	50
144	**E:J**	**EG4DMA:mMAOES**	47	56
145	**K:M**	**tBAEMA:EGDPEA**	47	74
146	**R:A**	**TDFOMA:HBOPBA**	46	77
147	**G:R**	**EGDA:TDFOMA**	45	106
148	**C:O**	**BHMOPhP:MAETA**	45	57
149	**Q:G**	**BA:EGDA**	45	53
150	**P:M**	**THFuA:EGDPEA**	45	42
151	**I:G**	**DEAMEA:EGDA**	44	37
152	**I:Q**	**DEAMEA:BA**	44	62
153	**M:L**	**EGDPEA:HEMA**	44	41
154	**C:G**	**BHMOPhP:EGDA**	44	40
155	**G:E**	**EGDA:EG4DMA**	43	51
156	**E:D**	**EG4DMA:TCDMDA**	43	47
157	**B:S**	**NGDA:HMAm**	42	71
158	**I:N**	**DEAMEA:FuMA**	42	44
159	**O:N**	**MAETA:FuMA**	41	51
160	**N:A**	**FuMA:HBOPBA**	41	37
161	**O:Q**	**MAETA:BA**	41	41
162	**H:L**	**GDMA:HEMA**	40	30
163	**E**	**EG4DMA**	40	49
164	**H**	**GDMA**	40	38
165	**E:S**	**EG4DMA:HMAm**	40	59
166	**F:S**	**BDDA:HMAm**	40	51
167	**G:B**	**EGDA:NGDA**	38	41
168	**Q:C**	**BA:BHMOPhP**	38	47
169	**L:G**	**HEMA:EGDA**	37	50
170	**P:A**	**THFuA:HBOPBA**	36	31
171	**B:E**	**NGDA:EG4DMA**	36	25
172	**O:P**	**MAETA:THFuA**	36	32
173	**N:B**	**FuMA:NGDA**	36	40
174	**A:J**	**HBOPBA:mMAOES**	35	45
175	**P:S**	**THFuA:HMAm**	35	28
176	**L:O**	**HEMA:MAETA**	34	47
177	**Q:A**	**BA:HBOPBA**	34	48
178	**R:H**	**TDFOMA:GDMA**	34	54
179	**M:N**	**EGDPEA:FuMA**	34	76

180	**D:S**	**TCDMDA:HMAm**	34	35
181	**K:A**	**tBAEMA:HBOPBA**	34	79
182	**H:J**	**GDMA:mMAOES**	33	58
183	**C:S**	**BHMOPhP:HMAm**	33	67
184	**E:H**	**EG4DMA:GDMA**	33	53
185	**R:D**	**TDFOMA:TCDMDA**	33	26
186	**L:I**	**HEMA:DEAEMA**	32	39
187	**N:P**	**FuMA:THFuA**	32	31
188	**K:B**	**tBAEMA:NGDA**	32	39
189	**K:E**	**tBAEMA:EG4DMA**	31	46
190	**P:O**	**THFuA:MAETA**	31	40
191	**O**	**MAETA**	31	15
192	**Q:O**	**BA:MAETA**	31	25
193	**B:A**	**NGDA:HBOPBA**	31	50
194	**B:P**	**NGDA:THFuA**	30	34
195	**K:L**	**tBAEMA:HEMA**	30	23
196	**R:N**	**TDFOMA:FuMA**	29	21
197	**K:P**	**tBAEMA:THFuA**	29	24
198	**Q**	**BA**	29	59
199	**E:O**	**EG4DMA:MAETA**	29	39
200	**O:S**	**MAETA:HMAm**	29	20
201	**C:I**	**BHMOPhP:DEAEMA**	28	57
202	**K:F**	**tBAEMA:BDDA**	28	38
203	**Q:R**	**BA:TDFOMA**	28	37
204	**S:F**	**HMAm:BDDA**	28	67
205	**Q:H**	**BA:GDMA**	27	48
206	**N:G**	**FuMA:EGDA**	27	32
207	**Q:F**	**BA:BDDA**	27	27
208	**A:I**	**HBOPBA:DEAEMA**	26	40
209	**N:L**	**FuMA:HEMA**	26	34
210	**K:Q**	**tBAEMA:BA**	26	23
211	**J:E**	**mMAOES:EG4DMA**	26	23
212	**E:Q**	**EG4DMA:BA**	26	46
213	**R:E**	**TDFOMA:EG4DMA**	25	24
214	**A:S**	**HBOPBA:HMAm**	25	30
215	**J:F**	**mMAOES:BDDA**	25	34
216	**C:Q**	**BHMOPhP:BA**	24	40
217	**K:C**	**tBAEMA:BHMOPhP**	23	25
218	**J:G**	**mMAOES:EGDA**	23	31
219	**A:N**	**HBOPBA:FuMA**	23	52
220	**P:J**	**THFuA:mMAOES**	22	25
221	**Q:N**	**BA:FuMA**	22	25
222	**I:C**	**DEAMEA:BHMOPhP**	22	27
223	**I:A**	**DEAMEA:HBOPBA**	21	20
224	**Q:M**	**BA:EGDPEA**	21	40
225	**A:M**	**HBOPBA:EGDPEA**	21	36

226	**E:K**	**EG4DMA:tBAEMA**	21	41
227	**R:P**	**TDFOMA:THFuA**	21	22
228	**I:K**	**DEAMEA:tBAEMA**	21	21
229	**I:M**	**DEAMEA:EGDPEA**	21	20
230	**C:N**	**BHMOPhP:FuMA**	20	25
231	**H:S**	**GDMA:HMAm**	20	26
232	**Q:P**	**BA:THFuA**	20	35
233	**E:M**	**EG4DMA:EGDPEA**	20	52
234	**L:S**	**HEMA:HMAm**	19	27
235	**S:G**	**HMAm:EGDA**	19	16
236	**G:M**	**EGDA:EGDPEA**	19	29
237	**D:K**	**TCDMDA:tBAEMA**	19	24
238	**C:H**	**BHMOPhP:GDMA**	19	21
239	**S:N**	**HMAm:FuMA**	18	17
240	**F:A**	**BDDA:HBOPBA**	18	16
241	**Q:L**	**BA:HEMA**	18	20
242	**P:L**	**THFuA:HEMA**	18	20
243	**G:S**	**EGDA:HMAm**	18	23
244	**M:P**	**EGDPEA:THFuA**	18	34
245	**J**	**mMAOES**	18	34
246	**P:I**	**THFuA:DEAEMA**	18	11
247	**S:E**	**HMAm:EG4DMA**	18	21
248	**J:A**	**mMAOES:HBOPBA**	17	23
249	**I:O**	**DEAMEA:MAETA**	17	16
250	**K:G**	**tBAEMA:EGDA**	17	19
251	**L:F**	**HEMA:BDDA**	17	24
252	**K:R**	**tBAEMA:TDFOMA**	17	13
253	**I:L**	**DEAMEA:HEMA**	17	14
254	**A:P**	**HBOPBA:THFuA**	17	34
255	**L:P**	**HEMA:THFuA**	17	21
256	**B:Q**	**NGDA:BA**	16	21
257	**E:I**	**EG4DMA:DEAEMA**	16	15
258	**R:M**	**TDFOMA:EGDPEA**	16	27
259	**K:N**	**tBAEMA:FuMA**	16	13
260	**I:R**	**DEAMEA:TDFOMA**	16	36
261	**R:J**	**TDFOMA:mMAOES**	16	19
262	**B:K**	**NGDA:tBAEMA**	16	18
263	**M:S**	**EGDPEA:HMAm**	16	23
264	**C:A**	**BHMOPhP:HBOPBA**	16	28
265	**M:G**	**EGDPEA:EGDA**	16	26
266	**R:S**	**TDFOMA:HMAm**	16	19
267	**E:N**	**EG4DMA:FuMA**	16	25
268	**S:D**	**HMAm:TCDMDA**	16	15
269	**L:C**	**HEMA:BHMOPhP**	16	20
270	**A:O**	**HBOPBA:MAETA**	15	28
271	**L:H**	**HEMA:GDMA**	15	26

272	**M**	**EGDPEA**	15	18
273	**K**	**tBAEMA**	15	8
274	**S:M**	**HMAm:EGDPEA**	15	7
275	**J:P**	**mMAOES:THFuA**	15	18
276	**K:H**	**tBAEMA:GDMA**	14	14
277	**G:I**	**EGDA:DEAEMA**	14	19
278	**R:K**	**TDFOMA:tBAEMA**	14	12
279	**G:H**	**EGDA:GDMA**	14	15
280	**A:Q**	**HBOPBA:BA**	13	23
281	**K:I**	**tBAEMA:DEAEMA**	13	10
282	**S:K**	**HMAm:tBAEMA**	13	27
283	**P:K**	**THFuA:tBAEMA**	13	23
284	**J:M**	**mMAOES:EGDPEA**	13	11
285	**B:N**	**NGDA:FuMA**	13	11
286	**O:R**	**MAETA:TDFOMA**	13	13
287	**R:O**	**TDFOMA:MAETA**	12	9
288	**G:K**	**EGDA:tBAEMA**	12	19
289	**R:L**	**TDFOMA:HEMA**	12	10
290	**R:Q**	**TDFOMA:BA**	11	13
291	**S:C**	**HMAm:BHMOPhP**	11	11
292	**L:N**	**HEMA:FuMA**	11	8
293	**R**	**TDFOMA**	11	11
294	**I**	**DEAEMA**	11	12
295	**O:L**	**MAETA:HEMA**	11	13
296	**G:L**	**EGDA:HEMA**	10	12
297	**S:O**	**HMAm:MAETA**	10	11
298	**C:P**	**BHMOPhP:THFuA**	10	9
299	**L:Q**	**HEMA:BA**	10	9
300	**N:R**	**FuMA:TDFOMA**	10	14
301	**N:M**	**FuMA:EGDPEA**	10	13
302	**N:S**	**FuMA:HMAm**	10	10
303	**K:D**	**tBAEMA:TCDMDA**	10	9
304	**S:J**	**HMAm:mMAOES**	9	7
305	**J:N**	**mMAOES:FuMA**	9	16
306	**J:C**	**mMAOES:BHMOPhP**	9	9
307	**I:J**	**DEAMEA:mMAOES**	9	10
308	**S:L**	**HMAm:HEMA**	9	7
309	**O:I**	**MAETA:DEAEMA**	9	16
310	**S:B**	**HMAm:NGDA**	9	8
311	**S:I**	**HMAm:DEAEMA**	9	5
312	**I:S**	**DEAEMA:HMAm**	9	8
313	**M:J**	**EGDPEA:mMAOES**	9	8
314	**C:M**	**BHMOPhP:EGDPEA**	8	12
315	**S:H**	**HMAm:GDMA**	8	6
316	**A:K**	**HBOPBA:tBAEMA**	8	13
317	**G:N**	**EGDA:FuMA**	8	7

318	**S:P**	**HMAm:THFuA**	8	14
319	**L:K**	**HEMA:tBAEMA**	8	6
320	**K:S**	**tBAEMA:HMAm**	8	6
321	**E:L**	**EG4DMA:HEMA**	8	16
322	**R:C**	**TDFOMA:BHMOPhP**	8	10
323	**J:K**	**mMAOES:tBAEMA**	8	7
324	**Q:K**	**BA:tBAEMA**	8	6
325	**S:A**	**HMAm:HBOPBA**	8	9
326	**Q:J**	**BA:mMAOES**	7	13
327	**L**	**HEMA**	7	6
328	**Q:S**	**BA:HMAm**	7	12
329	**O:K**	**MAETA:tBAEMA**	7	10
330	**R:I**	**TDFOMA:DEAEMA**	7	5
331	**A:L**	**HBOPBA:HEMA**	7	7
332	**O:J**	**MAETA:mMAOES**	6	9
333	**L:J**	**HEMA:mMAOES**	6	7
334	**E:R**	**EG4DMA:TDFOMA**	6	15
335	**F:Q**	**BDDA:BA**	6	8
336	**E:P**	**EG4DMA:THFuA**	6	9
337	**K:J**	**tBAEMA:mMAOES**	6	8
338	**F:N**	**BDDA:FuMA**	6	7
339	**N:O**	**FuMA:MAETA**	6	8
340	**G:P**	**EGDA:THFuA**	6	5
341	**Q:I**	**BA:DEAEMA**	6	5
342	**O:C**	**MAETA:BHMOPhP**	5	7
343	**J:H**	**mMAOES:GDMA**	5	8
344	**J:O**	**mMAOES:MAETA**	5	8
345	**L:R**	**HEMA:TDFOMA**	5	6
346	**S:R**	**HMAm:TDFOMA**	4	4
347	**N:J**	**FuMA:mMAOES**	4	6
348	**S:Q**	**HMAm:BA**	4	4
349	**G:Q**	**EGDA:BA**	4	5
350	**J:L**	**mMAOES:HEMA**	3	4
351	**J:S**	**mMAOES:HMAm**	3	6
352	**I:P**	**DEAMEA:THFuA**	3	3
353	**J:I**	**mMAOES:DEAEMA**	3	4
354	**D:R**	**TCDMDA:TDFOMA**	3	4
355	**J:Q**	**mMAOES:BA**	2	3
356	**J:R**	**mMAOES:TDFOMA**	2	3
357	**S**	**HMAm**	2	3
358	**C:K**	**BHMOPhP:tBAEMA**	2	1
359	**C:R**	**BHMOPhP:TDFOMA**	2	3

Table S4: Integrin blocking antibodies and peptides.

**Product Name**	**Cat. No.**	**Description**
**Integrin-blocking antibodies (R&D Systems)**		
Anti - α_2(CD49b)	MAB1233	Binds to α_2 integrin receptor
Anti - α_5(CD49e)	MAB1864	Binds to α_5 integrin receptor
Anti - α6 (CD49f)	MAB1350	Binds to α_6 integrin receptor
Anti - $\alpha_V\beta_3$	MAB3050	Binds to $\alpha_V\beta_3$ integrin receptor
Anti - $\alpha_V\beta_5$	MAB2528	Binds to $\alpha_V\beta_5$ integrin receptor
Anti - β_1	MAB17782	Binds to β_1 integrin receptor
**Integrin-blocking peptides (BACHEM)**		
H-1830	4009173	**RGD:** Linear peptide (Mw:346.35 Da)
H-4088	4027886	**c(RADfV):** Control peptide for H-2574.(Mw: 588.66 Da)
H-2574	4026200	**c(RGDfV)**: Binds $\alpha_V\beta_3$ and $\alpha_V\beta_5$ integrin receptors. (Mw: 574.64 Da)
H-7232	4070810	**C(RADfC):** Control peptide for H-7226. (Mw:592.68 Da)
H-7226	4069272	**c(RGDfC):** Binds $\alpha_V\beta_3$ integrin receptors. (Mw: 578.64 Da)
H-3164	4030598	**GRGDsP:** Inhibits binding to fibronectin. (Mw: 587.59 Da)
